# Environmental DNA can act as a biodiversity barometer of anthropogenic pressures in coastal ecosystems

**DOI:** 10.1038/s41598-020-64858-9

**Published:** 2020-05-20

**Authors:** Joseph D. DiBattista, James D. Reimer, Michael Stat, Giovanni D. Masucci, Piera Biondi, Maarten De Brauwer, Shaun P. Wilkinson, Anthony A. Chariton, Michael Bunce

**Affiliations:** 1 Trace and Environmental DNA (TrEnD) Laboratory, School of Molecular and Life Sciences, Curtin University, Perth, WA 6102 Australia; 2 Australian Museum Research Institute, Australian Museum, 1 William St, Sydney, NSW 2010 Australia; 3 Molecular Invertebrate and Systematics Ecology Laboratory, Graduate School of Engineering and Science, University of the Ryukyus, 1 Senbaru, Nishihara Okinawa, 903-0213 Japan; 4 Tropical Biosphere Research Center, University of the Ryukyus, 1 Senbaru, Nishihara Okinawa, 903-0213 Japan; 5 School of Environmental and Life Sciences, The University of Newcastle, Callaghan, NSW 2308 Australia; 6 School of Biological Sciences, Victoria University of Wellington, PO Box 600, Wellington, 6140 New Zealand; 7 School of Biology, Faculty of Biological Sciences, University of Leeds, Leeds, LS2 9JT United Kingdom; 8 Department of Biological Sciences, Macquarie University, North Ryde, 2113 Australia; 9 Environmental Protection Authority, 215 Lambton Quay, Wellington, 6011 New Zealand

**Keywords:** Biodiversity, Ecological genetics, Molecular ecology, Marine biology, Environmental impact

## Abstract

Loss of biodiversity from lower to upper trophic levels reduces overall productivity and stability of coastal ecosystems in our oceans, but rarely are these changes documented across both time and space. The characterisation of environmental DNA (eDNA) from sediment and seawater using metabarcoding offers a powerful molecular lens to observe marine biota and provides a series of ‘snapshots’ across a broad spectrum of eukaryotic organisms. Using these next-generation tools and downstream analytical innovations including machine learning sequence assignment algorithms and co-occurrence network analyses, we examined how anthropogenic pressures may have impacted marine biodiversity on subtropical coral reefs in Okinawa, Japan. Based on 18 S ribosomal RNA, but not ITS2 sequence data due to inconsistent amplification for this marker, as well as proxies for anthropogenic disturbance, we show that eukaryotic richness at the family level significantly increases with medium and high levels of disturbance. This change in richness coincides with compositional changes, a decrease in connectedness among taxa, an increase in fragmentation of taxon co-occurrence networks, and a shift in indicator taxa. Taken together, these findings demonstrate the ability of eDNA to act as a barometer of disturbance and provide an exemplar of how biotic networks and coral reefs may be impacted by anthropogenic activities.

## Introduction

Coastal ecosystems are biologically rich and provide resources for millions of people through fisheries, tourism, shipping, and resource extraction. An important component of coastal ecosystems in both tropical and subtropical environments are zooxanthellate scleractinian corals, a matrix of calcium carbonate skeleton and live animal polyps that provide habitat, food, and refuge, directly leading to high levels of marine biological diversity. In recent years, significant declines in coral habitat have been attributed to deteriorating coastal water quality^1^, anthropogenically induced climate change^2,3^, and coastal development^4^. These stressors also impede the ability of corals to compete for resources with other reef organisms such as macroalgae or sponges^1,5^, thereby causing compositional shifts in reef communities. For example, on degrading Caribbean reefs, phenotypically plastic coral genera such as *Porites*, *Siderastrea*, or *Agaricia* replaced more sensitive genera such as *Acropora* and *Orbicella*^6^. As another example, in the Red Sea near Eilat, disturbance to mesophotic scleractinian corals was hypothesised to allow the colonisation of other animals such as octocorals, tunicates, sponges, bryozoans, polychaetes, and molluscs on dead portions of their external skeleton^7^. These shifts in community structure often occur at small spatial scales, or over short periods of time, and these ephemeral changes can be difficult to detect.

Environmental DNA (eDNA) sampling in combination with next-generation sequencing (NGS) metabarcoding can provide an immediate option as a marine monitoring tool in both temperate^8–10^ and tropical^11–13^ environments, as well as areas of transition between the two^14,15^. This approach presents a rapid assessment of unicellular eukaryotes to resident small- and large-bodied animals by amplifying their DNA. In part, eDNA circumvents the need to recruit multiple taxonomic experts and the logistical constraints of exhaustive surveys of a large geographic area. That said, metabarcoding data have been largely restricted to size-fractionated plankton communities in the pelagic zone^16,17^ or based on material extracted from long-term (>1 year) benthic collection devices^18,19^. eDNA has therefore rarely been surveyed in a rapid and repeated manner or focused on multiple localised positions along reef and rocky coastlines.

We here chose to focus our surveys on coastal ecosystems in Okinawa (Ryukyu Archipelago, southern Japan), which is known for its high marine biodiversity, including several endemic taxa^20^. A total of 340 species of hard coral^21–23^ and at least 1200 species of fish^24,25^ have been documented from Okinawa, although much less is known about lower order invertebrate taxa^26^. The coastal areas of many islands in Okinawa are facing increasing anthropogenic pressures due primarily to coastal development and land reclamation projects^4,27–29^, but also due to the input of marine pollution including red soil runoff, endocrine disrupters, and excessive nutrients^30–33^. This human impact is mostly prevalent at the main island of Okinawa (land area: 1208 km^2^; population: ~1.3 million)^4,34^, with the southern portion of the island characterised by coral reef lagoons that have been reclaimed, high nutrient input from iron-rich terrestrial soil, and high fishing pressure. The Ryukyu Archipelago was also subjected to severe coral bleaching during the 1998 ENSO event^22^, and more recently from 2016 to 2017^35–37^, with branching and tabular hard coral taxa decreasing the most compared to stress-tolerant genera (e.g. *Porites*, *Dipsastraea*, *Favites*, and *Leptastrea*). Thus, while some aspects of the coral reefs of Okinawa have been well surveyed, there is a notable paucity of research focused on the wider biological impacts of these anthropogenic drivers^26^.

In this study, using (i) a ‘universal’ metabarcoding assay targeting 18 S ribosomal RNA (18 S rRNA) of most marine eukaryotes, as well as (ii) a more taxon-focused internal transcribed spacer 2 of ribosomal RNA (ITS2) assay targeting cnidarians and sponges, we investigated whether anthropogenic disturbances affected taxonomic diversity and richness. We additionally tested the connectedness and fragmentation among taxa across sites that experience different levels of anthropogenic pressures, and identified putative keystone taxa (i.e. highly connected nodes within networks). These data contribute to the growing body of eDNA literature and support the utility of metabarcoding in biological monitoring of our ocean ecosystems.

## Results

### Total biodiversity detected – 18 S rRNA

Using our ‘universal’ assay targeting the 18 S rRNA region, a total of 14,003,698 amplicon reads were generated from 42 sediment samples (2,945,188 sequences) and 164 seawater samples (11,058,510 sequences) to provide a snapshot of marine eukaryotic biodiversity along an anthropogenic pressure gradient at 14 sites in Okinawa, Japan (see Table S2). One sediment sample (now *N* = 41) and 31 seawater samples (now *N* = 133) failed to amplify, amplified poorly, or did not pass our sequencing depth threshold set at 20,000 reads. The number of assigned versus unassigned unique sequences per site ranged from 261 to 2114 (mean assigned ± SE, 1218 ± 157) or 389 to 2127 (mean unassigned ± SE, 1054 ± 131) sequences for sediment samples, and 701 to 7151 (mean assigned ± SE, 3658 ± 510) or 664 to 5904 (mean unassigned ± SE, 2646 ± 377) for seawater samples, respectively, when both years of data were combined.

**Figure 1:**
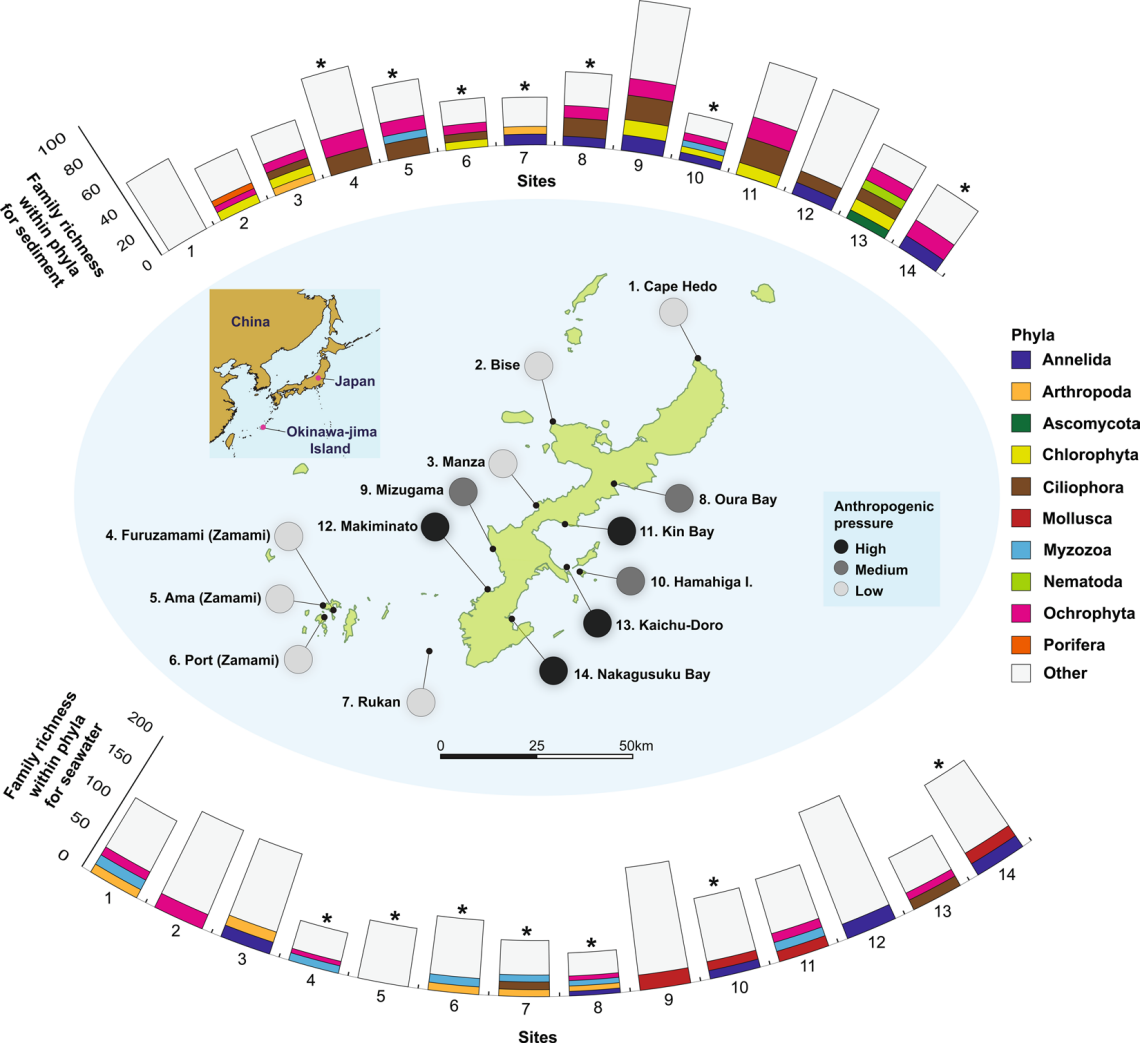
Sediment and seawater samples collected at 14 sites off the coast of Okinawa, Japan. Circles on the map are shaded according to the level of anthropogenic pressure that they experience (low pressure = light grey, medium pressure = intermediate grey, high pressure = dark grey). Bar graphs indicate the number of taxonomic families assigned at each site based on 18 S rRNA sequences; phyla where the number of families are greater than 10% of the total families for that site are coloured as indicated in the legend. An asterisk above bar graphs indicate sites that were sampled in one year only; sites without an asterisk were sampled twice, over two consecutive years. The figure was created with a combination of QGIS v 3.6 (https://www.qgis.org/en/site/) and Adobe Illustrator v CS6.

The 18 S rRNA metabarcoding data overall assigned to 39 eukaryotic phyla, 107 classes, 261 orders, and 498 families (Fig. 1, Table S2, Appendix S3). For sediment samples, families within the phyla Arthropoda and Porifera were only present (at > 10% of the total number of families per site) at low pressure sites, Annelida (with one site exception, i.e. Rukan) only appeared at medium and high pressure sites, and Ascomycota and Nematoda only appeared at high pressure sites. Similarly, for seawater samples, with one site exception (i.e. Oura Bay), families within the phylum Arthropoda only appeared (at > 10% of the total number of families per site) at low pressure sites, whereas families within the phyla Mollusca and, with one site exception (i.e. Cape Manza), Annelida only appeared at medium and high pressure sites.

### Family diversity (i.e. richness) analysis – 18 S rRNA

Based on 18 S rRNA metabarcode sequences, family diversity (i.e. richness) for sediment samples was 44.29 (±SE 4.69) for the low anthropogenic pressure sites, 59.00 (±SE 20.11) for the medium pressure sites, and 62.75 (± SE 6.92) for the high pressure sites (Fig. 1). For seawater samples, family diversity was 98.57 (±SE 12.37) for the low anthropogenic pressure sites, 108.00 (±SE 30.32) for the medium pressure sites, and 124.75 (±SE 21.20) for the high pressure sites (Fig. 1). There were no statistical differences in the family-level taxonomic richness among the three anthropogenic pressure categories for sediment samples (Kruskal-Wallis test: chi-square = 4.8427, df = 2, *P* = 0.089), but there were differences among seawater samples (Kruskal-Wallis test: chi-square = 18.819, df = 2, *P* < 0.0001), with richness at low pressure sites significantly lower than that at medium (Pairwise Wilcoxon Rank Sum test: *P* < 0.001) and high pressure (Pairwise Wilcoxon Rank Sum test: *P* < 0.001) sites; medium and high pressure sites were no different from each other (Pairwise Wilcoxon Rank Sum test: *P* = 0.88).

### Distinguishing community assemblages - 18 S rRNA

**Figure 2:**
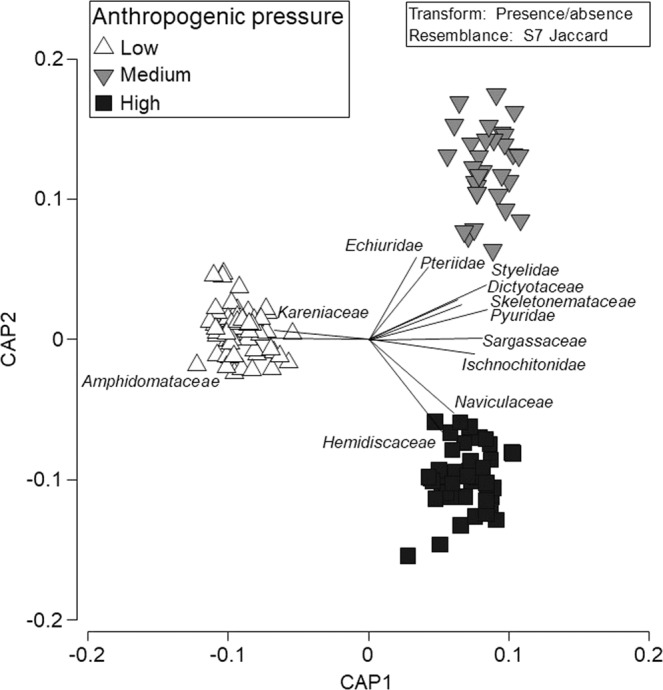
Canonical Analysis of Principle Coordinates (CAP) ordination plot of the presence/absence of eukaryotic families detected based on seawater samples collected at 14 sites in Okinawa, Japan and 18 S rRNA sequences. The relationship of eukaryotic community assemblages identified in each sample using a Jaccard’s coefficient for factor “Impact” is shown. Pearson correlation vectors (r > 0.4) represent the eukaryotic taxa driving the relationship among samples.

Based on 18 S rRNA metabarcoding sequences and PERMANOVA tests, although there was no significant effect of anthropogenic pressure on the assemblage of families recovered from sediment samples (*P* = 0.104, df = 2, MS = 5130, Pseudo-F = 1.2181), there was a significant effect for seawater samples (*P* = 0.0137, df = 2, MS = 15615, Pseudo-F = 1.732) (Fig. 2). Indeed, like family richness, the community assemblages at low pressure sites were different from medium pressure and high pressure sites, whereas medium and high pressure sites were no different from each other.

Based on follow-up IndVal analyses to resolve taxonomic families of importance in community classifications across anthropogenic pressure categories in sediment (Table 1), we identified chitons (family Chitonidae), free-living flatworms (family Macrostomidae), and sludge or sewage worms (family Naididae) as indicators of high pressure sites, and polychaete worms (family Capitellidae) as an indicator of medium and high pressure sites. For seawater samples, we identified siphonophores (family Agalmatidae), small dinoflagellates (family Ceratiaceae and Kareniaceae), siliceous haptophytes (family Prymnesiaceae), and demosponges (family Petrosiidae) as indicators of low pressure sites. We also identified marine algae (family Fragilariaceae and Rhodellaceae), unicellular flagellates (family Bicosoecidae), and marine slime molds (family Labyrinthula) as indicators of high pressure sites, as well as venus clams (family Veneridae), brown algae (family Dictyotaceae), marine algae (family Sargassaceae), a different group of demosponges (family Callyspongiidae), and haminoeid bubble snails (family Haminoeidae) as indicators of medium and high pressure sites.

**Table 1:** Indicator value (IndVal) analyses based on sediment and seawater samples collected at 14 sites in Okinawa, Japan and 18 S rRNA sequences for low, medium, and high anthropogenic pressure sites.

	Low pressure			Medium pressure			High pressure		
	Family	Common name	stat/P	Family	Common name	stat/P	Family	Common name	stat/P
Sediment				Capitellidae	Polychaete worms	0.89/0.050	Capitellidae	Polychaete worms	0.89/0.050
							Chitonidae	Chitons	0.87/0.022
							Macrostomidae	Free-living flatworms	0.87/0.016
							Naididae	Sewage worms	0.87/0.018
Water	Agalmatidae	Siphonophores	0.82/0.021	Veneridae	Venus clams	1.00/0.003	Veneridae	Venus clams	1.00/0.003
	Kareniaceae	Dinoflagellates	0.82/0.046	Dictyotaceae	Brown algae	0.93/0.017	Dictyotaceae	Brown algae	0.93/0.017
	Prymnesiaceae	Siliceous haptophytes	0.82/0.037	Sargassaceae	Marine algae	0.87/0.011	Sargassaceae	Marine algae	0.87/0.011
	Ceratiaceae	Siliceous haptophytes	0.80/0.049	Callyspongiidae	Demosponges	0.79/0.047	Callyspongiidae	Demosponges	0.79/0.047
	Petrosiidae	Demosponges	0.80/0.046	Haminoeidae	Bubble snails	0.79/0.029	Haminoeidae	Bubble snails	0.79/0.029
							Rhodellaceae	Marine algae	0.89/0.012
							Bicosoecidae	Unicellular flagellates	0.87/0.030
							Labyrinthula	Marine slime molds	0.87/0.025
							Fragilariaceae	Marine algae	0.84/0.035

P-values were selected at a 0.05 significance level.

### Network analyses

**Figure 3:**
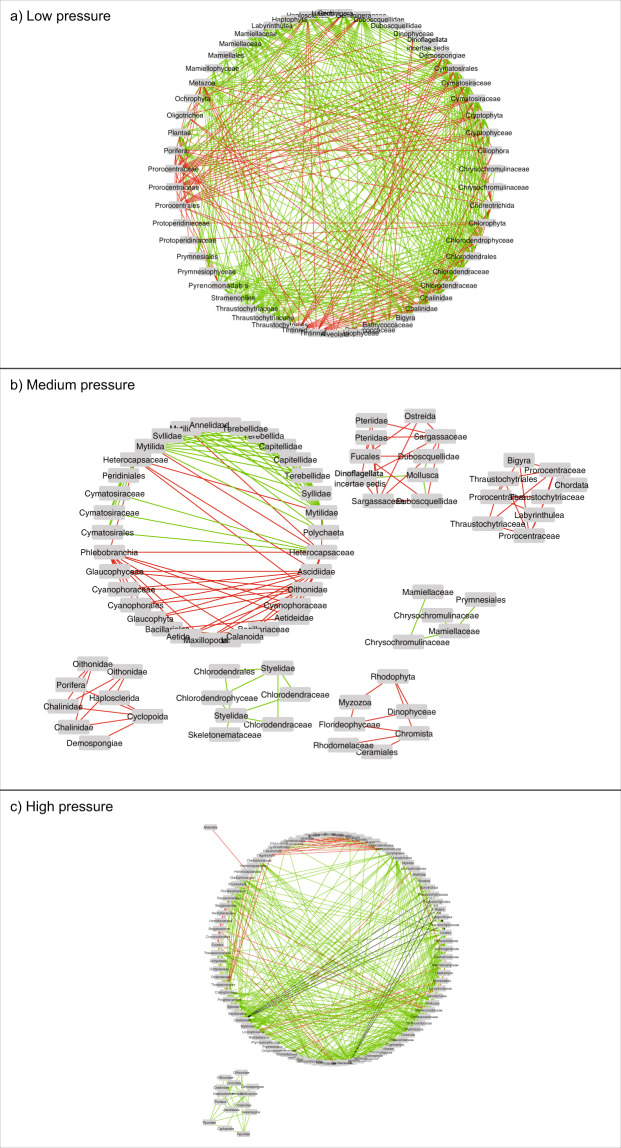
Co-occurrence networks of eukaryotic families detected based on seawater samples collected at 14 sites in Okinawa, Japan, and 18 S rRNA sequences for (a) low, (b) medium, and (c) high pressure sites. Green and red lines represent positive and negative interactions, respectively.

Visualisation of the three seawater networks based on sites experiencing low, medium, and high levels of anthropogenic pressure are provided in Fig. 3. The properties of each network and the top ten most connected nodes for each network are summarised in Tables 2 and 3, respectively. The number of nodes captured in each of three networks varied markedly, with the low pressure network having almost half (*N* = 55) the number of nodes compared to the high pressure network (*N* = 105). Despite the relatively lower number of nodes, the number of interactions as well as the proportion of positive and negative interactions was similar between the low pressure and high pressure networks. Interestingly, the medium pressure network had relatively few interactions, and in contrast to the other networks, most of these interactions were negative. Despite containing the fewest number of nodes, the low pressure network was the most densely populated with edges (clustering co-efficient = 0.344). Indeed, even with its elevated number of nodes, the high pressure network still contained a lower density of edges (clustering co-efficient = 0.165) than the low pressure network, indicating that fewer nodes can be reached via other nodes^38^.

**Table 2:** Network properties of the low, medium and high anthropogenic pressure co-occurrence networks based on presence/absence of eukaryotic families detected based on seawater samples collected at 14 sites in Okinawa, Japan and 18 S rRNA sequences.

Attributes	Low pressure	Medium pressure	High pressure
number of nodes	55	80	105
number of interactions	439	174	446
number of positive interactions	334	70	387
number of negative interactions	105	104	59
clustering co-efficient	0.344	0.066	0.165
connected components	1	6	2

**Table 3:** The ten most connected nodes from the low, medium and high anthropogenic pressure co-occurrence networks based on presence/absence of eukaryotic families detected based on seawater samples collected at 14 sites in Okinawa, Japan and 18 S rRNA sequences.

Pressure	Degree	Closeness centrality	Lineage
Low	30	0.6800	Stramenopiles–Ochrophyta–Bacillariophyceae–Cymatosirales–Cymatosiraceae
Low	30	0.8000	Stramenopiles–Ochrophyta–Bacillariophyceae–Cymatosirales
Low	30	0.9091	Stramenopiles–Ochrophyta–Bacillariophyceae–Cymatosirales–Cymatosiraceae
Low	29	0.8333	Metazoa
Low	26	0.6818	Plantae–Chlorophyta–Chlorodendrophyceae–Chlorodendrales–Chlorodendraceae
Low	26	0.6545	Plantae–Chlorophyta–Chlorodendrophyceae–Chlorodendrales
Low	26	0.7500	Plantae–Chlorophyta–Chlorodendrophyceae–Chlorodendrales–Chlorodendracea
Low	26	0.8571	Plantae–Chlorophyta–Chlorodendrophyceae
Low	25	0.6308	Metazoa–Porifera–Demospongiae–Haplosclerida–Chalinidae
Low	25	0.5698	Metazoa–Porifera–Demospongiae
**low average (all nodes)**	**16**	**0.5832**	
Medium	12	0.4714	Metazoa–Mollusca–Bivalvia–Mytilida–Mytilidae
Medium	12	0.4714	Metazoa–Mollusca–Bivalvia–Mytilida–Mytilidae
Medium	12	0.4714	Metazoa–Mollusca–Bivalvia–Mytilida
Medium	11	0.5000	Metazoa–Chordata–Ascidiacea–Phlebobranchia–Ascidiidae
Medium	11	0.5000	Metazoa–Chordata–Ascidiacea–Phlebobranchia
Medium	11	0.5000	Metazoa–Chordata–Ascidiacea–Phlebobranchia–Ascidiidae
Medium	9	0.5500	Chromista–Myzozoa–Dinophyceae–Peridiniales–Heterocapsaceae
Medium	9	0.5500	Chromista–Myzozoa–Dinophyceae–Peridiniales–Heterocapsaceae
Medium	8	0.3548	Metazoa–Annelida–Polychaeta–Phyllodocida
Medium	8	0.3548	Metazoa–Annelida–Polychaeta–Phyllodocida–Syllidae
**medium average (all nodes)**	**4**	**0.5135**	
High	40	0.5361	Plantae–Chlorophyta–Ulvophyceae–Ulvales–Ulvellaceae
High	39	0.5298	Plantae–Chlorophyta–Ulvophyceae–Ulvales
High	38	0.5235	Plantae–Chlorophyta–Ulvophyceae–Ulvales–Ulvellaceae
High	25	0.4611	Metazoa–Mollusca–Polyplacophora–Chitonida–Ischnochitonidae
High	24	0.4564	Metazoa–Mollusca–Polyplacophora–Chitonida–Ischnochitonidae
High	23	0.4279	Metazoa–Mollusca–Polyplacophora
High	23	0.4279	Metazoa–Mollusca–Polyplacophora–Chitonida
High	21	0.4198	Stramenopiles–Ochrophyta–Bacillariophyceae–Thalassiosirales–Thalassiosiraceae
High	21	0.4198	Stramenopiles–Ochrophyta–Bacillariophyceae–Thalassiosirales–Thalassiosiraceae
High	20	0.4428	Plantae–Chlorophyta–Ulvophyceae–Cladophorales
**high average (all nodes)**	**9**	**0.3676**	

The medium pressure network was weakly connected as indicated by its fragmentation into six components (Fig. 3); an additional small component as a result of fragmentation was also observed in the high pressure network. In contrast, the low pressure network consisted of single component. We additionally observed higher heterogeneities in both the medium and high pressure networks indicating a greater tendency for the creation of hub nodes. In general, the nodes from the low pressure network (mean number of neighbours = 15.96) were far more connected than those from both the medium (mean number of neighbours = 4.35) and high pressure (mean number of neighbours = 8.88) network.

The top ten putatively important taxa as determined by the highest degrees and closeness centrality varied among the networks (Tables 2 and 3). For the low pressure network, the important taxa identified included diatoms, chlorophytes from the order Cymatosirales, and demosponges. On average the low pressure nodes had a higher degree and higher closeness centrality versus those in the more disturbed networks. In the medium pressure network, these important nodes included mytillid bivalves, ascidians (order Phlebobranchia), dinoflagellates (family Heterocapsaceae), and polychaetes (order Phyllodocida). For the high pressure network, these important nodes included Ulvophyceae predominately from the order Ulvales, chitons (family Ischnochitonidae), and a different diatom from the low pressure network (Thalassiosirales, family Thalassiosiraceae).

### Anthozoa and Demospongiae richness and community composition analysis – ITS2

**Figure 4:**
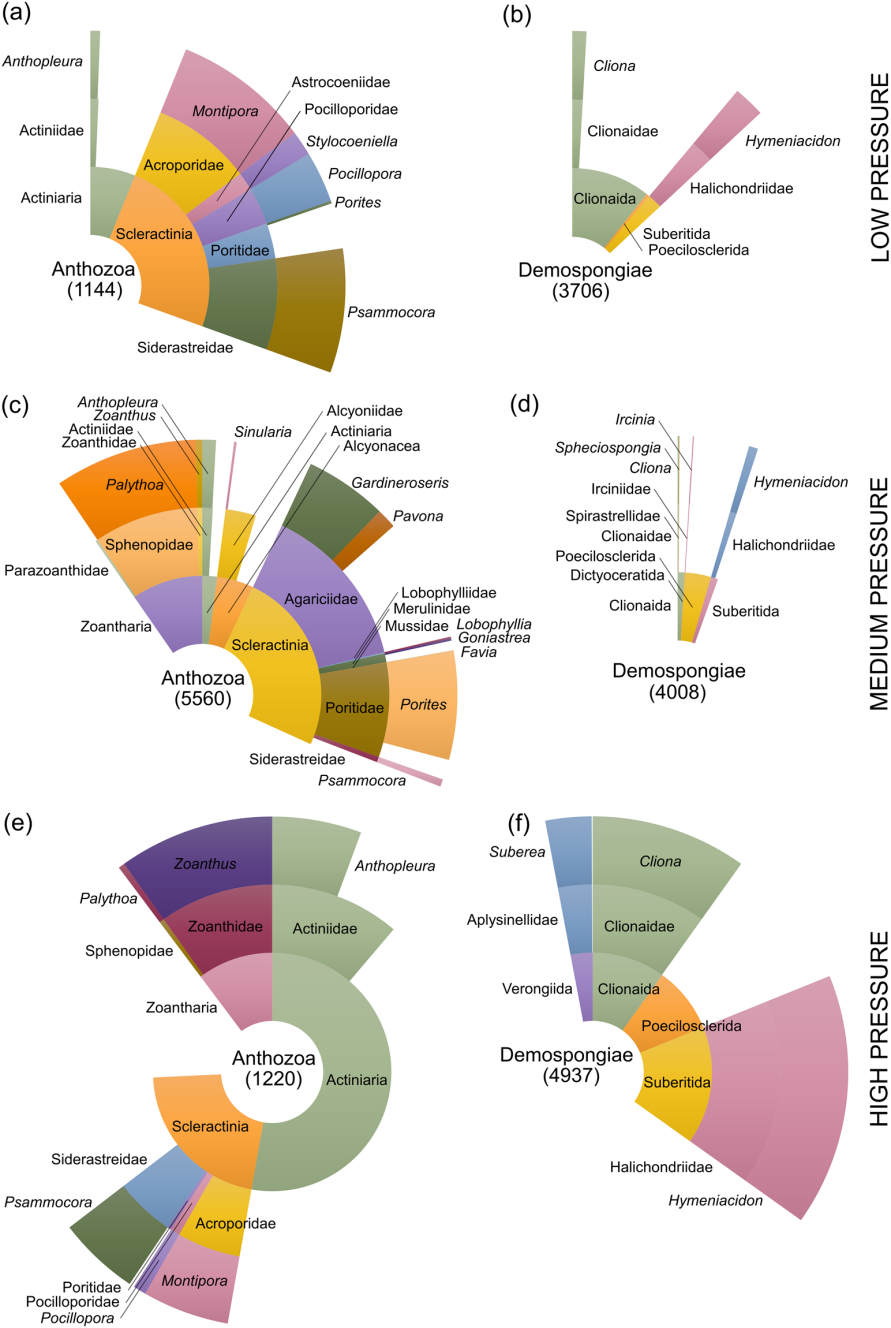
ITS2 sequences from seawater samples collected at low (**a,b**), medium (**c,d**), and high pressure sites (**e,f**) that were assigned to either class Anthozoa (left) or class Demospongiae (right) by the INSECT algorithm. Segment sizes and figures in parentheses are the absolute (i.e. not unique) number of sequences assigned at each taxonomic rank (order, family, and genus from inner to outer sections). Missing segments indicate the number of sequences unidentified at each rank.

A total of 4,220,102 ITS2 amplicon reads were generated from 26 sediment samples (2,261,264 sequences) and 71 seawater samples (1,958,838 sequences) (Table S1). Following subsampling, there were 13,776 unique sediment sequences and 4,213 unique seawater sequences from 200,000 and 44,000 total sediment and seawater reads, respectively. Of the unique sediment reads, 500 could be assigned to the class Anthozoa and 1,311 to Demospongiae; 11,184 sequences were not assigned and the remaining 781 sequences assigned to other eukaryotic taxa. Of the Anthozoa and Demospongiae, 262 could be assigned at the family level with confidence (five cnidarian families: Agariciidae, Merulinidae, Poritidae, Siderastreidae, and Sphenopidae), and these were distributed among only five of the ten sampling sites (Bise, Cape Hedo, Kaichu Doro S1, Makiminato, and Mizugama). Of the 4,213 unique reads subsampled from the seawater replicates, 772 could be assigned to the class Anthozoa and 1,161 to the class Demospongiae; 2,125 sequences were not assigned and the remaining 155 sequences assigned to other eukaryotic taxa. Of the Anthozoa and Demospongiae, 509 were assigned to 19 Anthozoa and Demospongiae families, with at least one ID positively assigned at all 11 sites. The most ubiquitous families were Poritidae (7 sites) and Halichondriidae (6 sites), whereas the families Agariciidae, Alcyoniidae, Aplysinellidae, Astrocoeniidae, Irciniidae, Lobophylliidae, Merulinidae, Mussidae, Parazoanthidae and Spirastrellidae were detected at only one site each (Fig. 4). Family richness for seawater ITS2 replicates was 3.6 (±SE 0.25) and 4.5 (± SE 2.23) for the low and high anthropogenic pressure categories, respectively. The increase in the mean and error for the latter was largely driven by the sampling site Mizugama, for which 15 families were detected. There were no statistical differences in the family-level taxonomic richness among the two anthropogenic pressure categories (Kruskal-Wallis test: chi-square = 0.86072, df = 1, *P* = 0.35), nor were there any differences in community composition detected between low and high anthropogenic pressure sites (*P* = 0.104, df = 2, MS = 5130, Pseudo-F = 1.2181).

## Discussion

We demonstrated that the biotic composition of marine taxa based on eDNA signatures in seawater was consistently transformed by anthropogenic pressure after controlling for the effects of spatial and temporal partitioning. We find that even though taxonomic richness increased with anthropogenic pressure based on seawater eDNA sampling (but not sediment eDNA sampling), it may not be reflective of compositional changes. Indeed, based on biotic community composition analyses, we found that sites that had been subjected to medium or high levels of anthropogenic pressure did not always lose keystone taxonomic groups, but instead may have replaced them with more opportunistic members within those groups. In fact, many of the indicator families were unicellular chlorophytes and diatoms, possibly due to higher turnover rates and sensitivity to environmental factors^39^, which stresses the necessity of focusing on a broad taxonomic spectrum to assess disturbance. Diatoms within the division Bacillariophyta are indeed one of the most important groups of microalgae given that they control 40–45% of primary production in our oceans^40^. Based on co-occurrence network analysis, we also found a decrease in connectedness and increase in fragmentation among the identified eukaryotic taxa, suggesting that disturbance may impact ecological functions and ecosystem resilience.

### Family diversity (i.e. richness) analysis – 18 S rRNA

Estimation of taxon richness based on families assigned from 18 S rRNA exhibited differences at small spatial scales among our study sites (a few to tens of kilometres) that were more likely attributed to anthropogenic pressures than abiotic variables, although we did not explicitly quantify the latter. Family-level richness went up with medium and high pressure, which suggests that surveys that rely exclusively on alpha-diversity as a proxy for impact, with higher diversity being deemed “good”, particularly for microbial communities^41^, may not capture the important compositional changes that can occur. One potential mechanism proposed for this increase in taxon richness or diversity in coastal ecosystems is that disturbances acting via bioerosion or the removal of existing species can provide microhabitat or create the space necessary for the recruitment of a different suite of species^7^. Indeed, albeit in soft-sediments, Ellis *et al*. (2017)^42^ found that the communities therein demonstrated the highest alpha-diversity in areas associated with mild disturbance. The “intermediate disturbance theory” also suggests that richness is the highest at intermediate levels of disturbance^43,44^. In such scenarios, rapid colonisers and more competitive species can co-occur. Chariton *et al*. (2015) found eDNA derived taxonomic richness to be consistently higher in anthropogenically influenced estuaries, with the authors suggesting that this was likely due to a confluence of inputs being deposited in these systems^14^. Recent studies based at offshore gas platforms in the North Adriatic Sea revealed that richness of benthic and planktonic eukaryotes however obtained from water samples did not show a clear pattern along a distance gradient from the putative source of disturbance^45^. Collectively, the evidence suggests that high eDNA derived taxonomic richness may not necessarily be indicative of a healthy environment. Indeed, variation in alpha-diversity is often non-linear and difficult to directly compare between studies given differences in methodology^41,46^. We strongly recommend always combining taxon richness metrics with an examination of the biotic composition, in addition to co-occurrence network analyses that can identify breakdowns in the cohesiveness of the community (see below).

### Distinguishing community assemblages - 18 S rRNA

It is established that different substrates will give a different picture of the marine environment based on the taxa that are recovered with eDNA metabarcoding^9^. In our case, the community composition of taxa recovered from sediment versus seawater did not overlap (Fig. S1), and so these were considered in isolation of each other for most of the analyses. In terms of biotic assemblages detected in the sediment, we found that animals from the phyla Arthropoda (e.g. horseshoe crabs, sea spiders, crustaceans) and Porifera (i.e. sponges) largely disappeared with anthropogenic pressure and were replaced by animals from the phyla Annelida (segmented worms) and Nematoda (marine round worms), although these shifts were not significant overall. We also saw an increase in worms of the family Naididae at high pressure sites, which are considered an indicator of low water quality^47^. Nematodes account for approximately 80% of all individual animals on earth^48^ and are characterised by high abundances in meiofaunal assemblages^49^. The fact that nematodes only appeared at high pressure sites in our study may reflect preferential amplification of other taxa present at less impacted sites^50^ or a shift from k-selected to r-selected taxa that is often associated with pollution^51^. We additionally identified a specific family of sediment-associated polychaete worm (Capitellidae) indicative of medium pressure and high pressure sites; these organisms have previously been recommended as proxies for environmental disturbances^52^. The lack of sensitivity of sediment sampling versus seawater sampling is consistent with previous work^9,53^, and may be due to the legacy of DNA in different substrates. For instance, eDNA does not last as long in the water column as it does in the sediment, which means the latter substrate captures a greater time window. This supports the idea that rapid community turnover may be easier to detect in the water column.

In terms of the biotic assemblages in seawater, we found that animals from the phylum Arthropoda disappeared with anthropogenic pressure and were replaced by animals from the phyla Annelida and Mollusca (Fig. 1). Low pressure sites were dominated by hydrozoans (family Agalmatidae), dinoflagellates (family Kareniaceae and Ceratiaceae), and demosponges (family Petrosiidae), whereas marine slime molds (family Labyrinthulidae), bubble snails (family Haminoeidae), venus clams (Veneridae), and a different group of demosponges (family Callyspongiidae) were putative indicators of medium pressure or high pressure sites. Some demosponges, including species of the Callyspongiidae family, are hypothesised to have negative effects on neighbouring scleractinian corals via the toxic compounds that they produce^54^. This same group of sponges are also hypothesised to dominate coral reef ecosystems under future climate change scenarios^55^.

### Network analyses

We found that the broad topological features of co-occurrence networks based on 18 S rRNA taxonomic assignments in seawater samples differed considerably among the three levels of anthropogenic pressure. We detected the presence of multiple independent components and a lower clustering coefficient in the higher-pressure categories, which led to fracturing of the networks^56,57^. This shift was particularly evident in the medium pressure network, which was also dominated by negative (instead of positive) taxon interactions. This shift also indicates a markedly different behaviour where amensalism and/or competition were driving the interactions between nodes, rather than commensalism and mutualism interactions observed in the low and high pressure networks. Although experimental evidence is limited, this is important because it has been suggested that an increase in positive inter-taxa interactions may improve the resilience of an ecosystem to stressors^56^. Several disturbance studies have shown similar reductions in the degree of links observed within networks, and this downward shift supports the potential utility of endpoints as metrics for detecting changes in co-occurrence networks^58–61^.

The networks identified a range of hub nodes (i.e. those that were highly connected) in both low and high pressure networks, indicating that they were intrinsically linked via numerous positive interactions. Interestingly, shifts in the taxonomy of the diatom nodes towards a more integrated role of Thalassiosiraceae as a response to anthropogenic pressures was also observed by Chariton *et al*. (2015) in Australian estuarine sediments^14^. Furthermore, using a Bayesian network, Graham *et al*. (2019) predicted a shift towards an increasing dominance of dinoflagellates, and indeed, this group was also observed within key nodes for the medium pressure network^62^. Surprisingly, many of the nodes identified in the medium and high pressure networks were associated with benthic species (e.g. spionid polychaetes, chitons, and mytillids). One possible explanation for this finding is that there is a distribution of the larval form of these animals in the water column. For example, spionids and mytillids are known to include broadcast spawners with planktotrophic or lecithotrophic larval stages^63,64^. The alternative is that DNA from these organisms is capable of mixing in the water column and intermittently becomes suspended as plankton.

While there are number of caveats associated with interpretation of the networks, given the spatial and temporal nature of which samples were collected, it does provide an additional line of evidence for understanding how communities respond to disturbances. Both theoretical and empirical studies of the relationship between diversity and ecosystem stability have generally found a positive association between the two, resulting in increased ecosystem resilience to external forces and greater support of ecosystem services^65,66^. However, as illustrated in this study and in Chariton *et al*. (2015), metabarcoding diversity may indeed be enriched in disturbed systems, suggesting that richness and diversity may be a more appropriate indicator of condition^14^.

A surprising result was the extent of fracturing and lower cohesion in the medium pressure network. One possible explanation is that these systems are in a state of flux and susceptible to increased negative interactions based around competition and parasitism^57^. In contrast, the shift from the low pressure to high pressure networks may be reflective of hysteresis, where the ecological communities are relatively stable in undisturbed or highly disturbed ecosystems, albeit founded on different nodes (i.e. taxa)^67^. While the fundamental hypothesis that connectivity is intrinsically linked to resilience is still being debated, the greater level of overall connectivity at both the network and key node scale suggest that low pressure systems have a greater level of built in redundancy, enabling composition turnover to occur without a loss of key biotic interactions.

### Anthozoa and Demospongiae richness and community composition analysis – ITS2

Our ITS2 metabarcoding assay focused on two important functional groups in coral reef ecosystems, animals from the classes Anthozoa and Demospongiae. Despite no statistical differences in the family-level taxonomic richness or community composition among two anthropogenic pressure categories considered here (low pressure and high pressure only due to small sample sizes), there were some striking qualitative patterns (Fig. 4). For class Anthozoa at the genus level, we noted an increasing trend in the proportion of sequences with anthropogenic pressure for *Anthopleura* (speciose genus of sea anemones), *Palythoa* (sand-encrusted colonial anemone), and *Zoanthus* (non-sand-encrusted colonial anemone). This trend is important because recent phase shifts from scleractinian hard corals to other anthozoan groups such as corallimorpharians^68^ and zoantharians such as *Palythoa*^69,70^ and *Zoanthus*^71^ have been reported, although often the underlying causes for their increases are unclear.

For class Demospongiae at the genus level, we noted an increase with anthropogenic pressure in the proportion of sequences for *Cliona* (speciose genera of sponge), *Hymeniacidon* (includes mobile sponge species), *Ircinia* (speciose genera of sponge), and *Suberea* (horny sponges). We also noted the highest diversity, or at least the highest proportion of successfully identified taxa at Mizugama, with 15 different Anthozoa or Demospongiae families present at this site. In a previous study, Mizugama was shown to be the only site out of eight investigated around Okinawa Island that was dominated by hard corals and *Palythoa* (family Sphenopidae)^69^, which is consistent with our observations here. That said, successful amplification for ITS2 was weak overall (Table S1), and a thorough evaluation would require additional sampling or sequencing coverage using this assay.

### Caveats

We have confidence in our 18 S rRNA metabarcoding findings and the repeatability of our assays given the grouping of replicates together from the same site, and the approximate grouping of site replicates together sampled in different years (Fig. S2). However, there are still important caveats of the eDNA metabarcoding method and therefore our data set as presented here. First, a significant fraction of our 18 S rRNA sequences post-filtering could not be assigned at the family level (19–63% of unique reads; Table S1). This deficiency reinforces the need for both improved DNA reference databases and a robust taxonomic framework. The INSECT algorithm^72^ used for ITS2 data, on the other hand, assigns taxon ID to sequences from complex environmental samples using hidden Markov models, and is designed to minimize false discoveries that persist in *k*-mer based assignment methods^73^. However, while misclassifications and over-classifications are generally rare, INSECT often under-classifies (i.e. does not assign an ID) if the reference database is underpopulated^72^. This, once again, stresses the need for the continued development of curated genetic databases representing multiple genes based on taxonomically identified specimens. Such initiatives can be supplemented with algorithms that can simultaneously minimize both type I and type II assignment errors. The present study, and others following similar approaches, do not include abiotic data in the analyses. We recommend collecting environmental data together with genetic material to provide a more robust explanation of changes in marine biological communities. Long-term collection of environmental samples *in situ* is preferred to remote sensing approaches, when feasible, given the difficulty of “scaling down” with the latter.

## Conclusions

Taken together this study adds to the growing body of literature that shows the utility of eDNA in providing a better understanding of marine environments. Even in its current state of development, with large apparent gaps in DNA reference databases, eDNA can act as a powerful method that complements existing survey methods. According to the results, 18 S provided a better understanding of the response by biological communities versus the assay targeting ITS2. This suggests a focus on developing the former versus the latter if a multi-assay approach is not possible based on limited funds. That said, the ITS2 assay has yet to be fully tested given insufficient sequencing coverage in this study. As marine biomonitoring increasingly moves towards a ‘ecosystem-based’ approach to track anthropogenic impacts these metabarcoding data support the ability of eDNA to deliver a more holistic survey of biota and identify indicator taxa. This aligns with new initiatives related to marine monitoring, and may additionally provide a standardized tool, outlined by a recent UN‐sponsored report by the Intergovernmental Science-Policy Platform on Biodiversity and Ecosystem Services (IPBES). Moreover, the continuation of temporal and spatial sampling with sufficient replication for more nuanced co-occurrence network analyses should further enrich the survey of both pristine and degraded marine environments across the globe.

## Materials and Methods

### Sampling sites and anthropogenic pressure scale

The selected sampling sites in Okinawa were differentially impacted by natural and anthropogenic pressures (Fig. 1). Although environmental data are available for the coastal ecosystems of this region, including sea surface temperature (SST; see Japan Meteorological Agency, https://ds.data.jma.go.jp/tcc/tcc/products/elnino/cobesst/cobe-sst.html), wave height (see Japan Meteorological Agency, https://www.data.jma.go.jp/gmd/kaiyou/db/wave/chart/daily/coastwave.html?year=2019&month=3&day=4&hour=12), additional water parameters (see Okinawa Prefecture, https://www.pref.okinawa.jp/site/kankyo/hozen/mizu_tsuchi/water/public_water.html), and for some areas, live coral cover (see Japan Ministry of Environment http://www.biodic.go.jp/trialSystem/top_en.html and^4^), there are not comprehensive or detailed enough to allow for the assessment of relative anthropogenic pressures at the geographic resolution we wished to examine (e.g. <2 km). Accordingly, we adopted a point-based assessment system in order to rapidly rank sampling sites based on cumulative anthropogenic impacts. Each of the sites was initially allotted 10 points, and points were then subtracted from this total based on *in situ* observations and publicly available information. The criteria considered and references justifying these guidelines were:

1. Distance from shore: -1 point if the sampling site was less than 5 km from the shoreline^74^;
2. Distance from heavily populated areas: -1 point if >10,000 people lived within 5 km of the sampling site^74^;
3. Freshwater input: -0.5 points if there was a natural river within 2 km of the sampling site; -1 point if there was treated wastewater input within 2 km of the sampling site^74^;
4. Fishing pressure: -1 point if there was significant fishing pressure (high number of fishing boat visits, shore fishing, and evidence of lost fishing lines, lures, and weights^75^);
5. Coastal development: -1 point if there was recent (<10 years) coastal development, including reclaimed land, “coastal defence” tetrapod placement, road construction, artificial reefs, dredging, or any other major alterations in the immediate vicinity; -0.5 points if there was less recent (>10 years ago) coastal development;^28,29^
6. Human recreation area: -1 point if the sampling site was adjacent to a human recreational area, including artificial beaches and frequent scuba diving or snorkeling locations;^76,77^
7. Hermatypic coral cover: -1 point if coral cover was between 0% to 25% at ~8 metres depth, -0.5 points if between 25% to 50%^78^;
8. Other notable pressures: -1 point if there was evidence of eutrophication, loss of water flow, pollution, or the site was dominated by macroalgae^79^.

To increase replication, sites were grouped by final scores into low anthropogenic pressure (scores >7), medium anthropogenic pressure (scores >4 but <7), and high anthropogenic pressure (scores <4) treatment groups.

### Sample collection and DNA extraction

Sampling was conducted in July 2016 and October 2017 at several sites around Okinawa (Fig. 1). For each sampling site, eight 1 L seawater replicates were collected from 30 cm below the surface using sterile Nalgene bottles. All water samples were immediately stored on ice and filtered at the University of the Ryukyus or in the field within eight hours. 750 ml of each water sample was filtered onto 47 mm polyethersulfone filters with a 0.2 $\mu$m pore size (Pall Life Sciences, New York, USA) using a Sentino peristaltic pump (Pall Life Sciences). The filtration apparatus was cleaned by soaking in 10% bleach between samples for at least 15 minutes. Approximately 10 g of marine sediment (up to four replicates per sampling site) was also collected with sterile 15 ml falcon tubes between 1 and 10 m depth snorkeling or on scuba. The falcon tubes remained closed until the moment of sampling and were closed immediately after sampling. Sediment samples were immediately frozen and stored at -20 °C until DNA extraction in a PCR-free extraction laboratory at Curtin University in Australia.

DNA bound to filter membranes was extracted using Qiagen DNeasy Blood and Tissue kits (Qiagen; Venlo, Netherlands) that was partially automated on a Qiacube (Qiagen) to minimise human handling and cross-contamination. Total nucleic acids were extracted from sediment samples following homogenization of 0.5 g to 0.8 g of organic material per replicate using bead tubes mixed on a Minilys homogenisation machine (Bertin Technologies, France); homogenised replicates were transferred into sterile 2 ml microfuge tubes. Due to the co-purification of inhibitors in sediment samples, a MoBio Powersoil extraction kit (MoBio Laboratories, CA) was used following the manufacturers protocol. A 2× volume of the homogenate was used at the digestion stage and the DNA was eluted in 100 µl of sterile elution buffer. DNA extraction controls (blanks) were carried out for every set of extractions and the appropriate level of input DNA for metabarcoding was determined by qPCR (see below).

### Fusion-tag qPCR

A universal primer set targeting 18 S rRNA (V1-3 hypervariable region; 18S_uni_1F: 5′ - GCCAGTAGTCATATGCTTGTCT - 3′; 18S_uni_400R: 5′ - GCCTGCTGCCTTCCTT - 3′) with an amplicon length of ~340-420 bp was used to maximise the eukaryotic fraction of marine diversity detected^80^. These data were fed into the taxonomy-dependent family-level richness and assemblage analyses outlined below. We used a second assay targeting ITS2 of Cnidaria and Porifera (scl58SF: 5′ - GARTCTTTGAACGCAAATGGC - 3′; scl28SR: 5′ - GCTTATTAATATGCTTAAATTCAGCG - 3′**)** to increase the taxonomic resolution of Anthozoa and Demospongiae detected within each environmental sample^81^. The former class includes anemones, stony corals, soft corals, and gorgonians, whereas the latter class includes 81% of all sponge species. Both groups play an important role in the functioning of coral reef ecosystems, such as recycling dissolved organic matter^82^.

Quantitative PCR (qPCR) experiments were set up in a separate ultra-clean laboratory at Curtin University designed for ancient DNA work using a QIAgility robotics platform^13^ (Qiagen Inc., CA). In brief, fusion-tag qPCR was performed in duplicate to mitigate reaction stochasticity on a StepOnePlus Real-Time PCR System (Applied Biosystems, CA, USA) under the following conditions for 18 S: initial denaturation at 95 °C for 5 min, followed by 45 cycles for 30 s at 95 °C, 30 s at 52 °C, and 45 s at 72 °C, with a final extension for 10 min at 72 °C. PCR was performed under the following conditions for ITS2: initial denaturation at 95 °C for 5 min, followed by 45 cycles of 30 s at 95 °C, 30 s at 55 °C, and 45 s at 72 °C, with a final extension for 10 min at 72 °C. Duplicate reactions were combined into a larger library of amplicons by pooling at equimolar ratios based on qPCR C_T_ values and the endpoint of amplification curves. These larger libraries were size-selected using a Pippin-Prep (Sage Science, Beverly, USA) to remove any amplicons outside of the target range. Size-selected libraries were then purified using the QIAquick PCR Purification Kit (Qiagen Inc.) and quantified against DNA standards of known molarity on a LabChip GX Touch (PerkinElmer Health Sciences, MA). Next-generation sequencing was conducted on an Illumina MiSeq platform (Illumina, San Diego, CA) housed in the Trace and Environmental DNA (TrEnD) Laboratory at Curtin University, Western Australia.

### Bioinformatic filtering

All sequence data were quality filtered (QF) prior to taxonomic assignment using taxonomy-dependent workflows. Metabarcoding reads recovered by paired-end sequencing were first stitched together using the Illumina MiSeq analysis software (MiSeq Reporter v 2.5) under the default settings. Sequences were then assigned to samples based on their unique index combinations and trimmed in Geneious Pro v 4.8.4^83^. In order to eliminate low quality sequences, only those with 100% identity matches to Illumina adaptors, index barcodes, and template specific oligonucleotides were kept for downstream analyses. Sequences were further processed in USEARCH v 9.2^73^, which was used to trim ambiguous bases, remove sequences with average error rates >1% and those that were <200 bp in length, dereplicate each sample down to unique sequences, abundance filter the unique sequences (minimum of two identical reads)^13^, and remove chimeras. To enable comparison among replicates, the 18 S sequences in each replicate were sub-sampled to 20,000 reads prior to dereplication. Due to lower amplification success of the ITS2 marker, replicates within sites were merged and subsampled to 20,000 and 4,000 reads prior to dereplication for sediment and seawater samples, respectively.

### Taxonomic assignment

Unique 18 S sequences that passed QF were queried against the National Center for Biotechnology Information (NCBI) nucleotide database using BLASTn and a high-performance computer (Magnus) located at the Pawsey Supercomputing Centre in Western Australia. BLASTn results were imported into MEtaGenome ANalyzer (MEGAN) v 5.11.3^84^, and taxonomic identities assigned at the family-level based on the lowest common ancestor (LCA) algorithm (minimum bit score = 600; top percent of reads = 5%; max expected = 0.01). Rarefaction analyses were performed in MEGAN v 5.11.3, and all taxonomic nomenclature was based on the World Register of Marine Species^85^, MycoBank, UniProt, and Algaebase. ITS2 sequences that passed QF were identified to family-level (or lower) where possible using the ‘insect’ R package v 1.3.0^72^ with the cnidarian_ITS2_marine classifier v 5. This classifier was trained on 8154 cnidarian, sponge, and other ITS2 reference sequences downloaded from NCBI GenBank on 20 Sep 2018 (10.5281/zenodo.3247241). Sensitivity settings were as follows: threshold = 0.8, decay = FALSE, ping = 0.99, mincount = 2, offset = 0. Sequences that were not identified as Anthozoa or Demospongiae were subsequently removed from the analyses.

### Network construction and analyses

To examine the connections between 18 S rRNA family-level taxon assignments identified in seawater samples collected from sites under varying levels of anthropogenic pressure, irrespective of year, co-occurrence networks were constructed using CoNet v 1.1 (http://systemsbiology.vub.ac.be/conet)^86^. Networks were derived by grouping all replicate samples into their appropriate anthropogenic pressure category (low, *N* = 60; medium, *N* = 31; high, *N* = 42). Families that were identified in less than 25% of the samples were discarded prior to computation and pairwise scores were determined using five similarity measures (Bray Curtis dissimilarity, Kullback‐Leibler dissimilarity, Pearson correlation, Spearman correlation, mutual information similarity). For each measure, 1000 renormalized permutation and bootstrap scores were generated^86^. Measure-specific P-values were merged using Brown’s method^87^, with correction for multiple-testing performed using the Benjamini–Hochberg procedure. Only network edges that agreed for all measures and resulted in the same interaction type (i.e. positive or negative) were retained. Co-occurrences were not produced using the sediment samples (low pressure, *N* = 17; medium pressure, *N* = 10; high pressure, *N* = 14) or the ITS2 data due to the relatively low number of replicates and limited representation of families across multiple samples. Indeed, the number of samples used to produce co-occurrence networks has a pronounced effect on the reliability of the network’s features^88^, with Reshef *et al*. (2011)^89^ recommending that at least 20 replicates is required to produce a network.

Visualisation of the networks was performed using Cytoscape v 3.7 (http://apps.cytoscape.org/apps/conet)^90^. The topographical features of the networks were examined using Network Analyzer v 2.7^91^, which included the number of nodes, the number of total positive and negative interactions, the clustering co-efficient (i.e. measure of the degree to which nodes form interactive modules)^38^, the number of modules, and the average number of neighbours^91^. Putative hub nodes (i.e. nodes that play a key role in network organisation) were determined by examining the top ten most connected nodes within each network^92^. In addition, the closeness centrality of the hub nodes was estimated based on the reciprocal of the sum of distances to all other nodes. Collectively, this information provided an indirect test of ecological redundancy (e.g. the “insurance hypothesis”)^93^, whereby if multiple taxa in an ecosystem perform similar tasks one taxon can replace the role of another.

### Statistical analyses

Taxonomic composition of marine eukaryotes at the family-level for 18 S was analysed in presence/absence format (Jaccard similarity matrix) to assess the effect of anthropogenic pressure (low, medium, high) and year (2016, 2017) on biological assemblages using PRIMER v 7^94^. Initial PERMANOVA tests showed that the community assemblage recovered with each method was significantly different (*P* < 0.0001, df = 1, MS = 43340, Pseudo-F = 12.003), resulting in a lack of overlap for families detected with each substrate (Fig. S1). Data from both methods (sediment versus seawater) were therefore analysed in isolation unless otherwise noted. Initial PERMANOVA tests showed that the community assemblage recovered from each year was not significantly different (*P* = 0.138, df = 1, MS = 11889, Pseudo-F = 1.9556), and so these data were combined for downstream analyses. PERMANOVA (9999 permutations) was therefore used to quantify the differences between anthropogenic pressure categories, and constrained Canonical Analysis of Principal coordinates (CAP) was used to visualise the hypothesis that assemblages detected at differently impacted locations were not the same. Indicator value (IndVal) analyses^95^ were also conducted using the Indicspecies package (v 1.7.8) in R^96,97^ to determine the eukaryotic families of importance in community classifications when compared among anthropogenic pressure categories. Family richness among pressure categories was compared using Kruskal-Wallis (KW) tests in R v 3.5.3^97^. Post-hoc pairwise rank sum Wilcoxon tests were used to compare between each pressure category.

The seawater family-level assignments of Anthozoa or Demospongiae ITS2 sequences were analysed in presence/absence format to assess the effect of anthropogenic pressure on community composition and richness using the vegdist (method = “jaccard”) and adonis (permutations = 9999) functions in the vegan R package (v 2.5–6)^97,98^, and the Kruskal-Wallis test in R v 3.5.3^97^. The effects of anthropogenic pressure on community assemblages were tested at two levels for the seawater ITS2 analysis (low pressure versus high pressure, with a score of 5 acting as the threshold) instead of three levels (low, medium, high) due to the relatively low number of sites that passed the subsampling threshold for sequencing depth (e.g. seawater samples from only ten sites yielded at least 4,000 ITS2 sequences). Moreover, taxonomic assignment of the sediment ITS2 samples failed to identify sufficient Anthozoa and Demospongiae sequences to assess the impact of anthropogenic pressure; only five cnidarian families were identified in total (Agariciidae, Merulinidae, Poritidae, Siderastreidae, and Sphenopidae), and only five sites yielded at least one Anthozoa or Demospongia family-level identification (see Results).

## Supplementary information

Supplementary information.

Supplementary information2.

Supplementary information3.

Supplementary information4.

Supplementary information5.

## Data Availability

All data needed to evaluate the study are available from Dryad Digital Repository 10.5061/dryad.m37pvmczf.
