# Supplementary material for: Environmental DNA can act as a biodiversity barometer of anthropogenic pressures in coastal ecosystems
Source: Sci Rep. 2020 May 20;10:8365. doi: 10.1038/s41598-020-64858-9 (PMC7239923; doi:10.1038/s41598-020-64858-9)

**Environmental DNA can act as a biodiversity barometer of anthropogenic pressures in coastal ecosystems**

**Joseph D. DiBattista^1,2*^, James D. Reimer^3,4^, Michael Stat^1,5^, Giovanni D. Masucci^3^, Piera Biondi^3^, Maarten De Brauwer^1,6^, Shaun P. Wilkinson^7^, Anthony A. Chariton^8^, Michael Bunce^1,9^**

Author affiliations:

^1^Trace and Environmental DNA (TrEnD) Laboratory, School of Molecular and Life Sciences, Curtin University, Perth, WA, 6102, Australia

^2^Australian Museum Research Institute, Australian Museum, 1 William St, Sydney, NSW, 2010, Australia

^3^Molecular Invertebrate and Systematics Ecology Laboratory, Graduate School of Engineering and Science, University of the Ryukyus, 1 Senbaru, Nishihara, Okinawa, 903-0213, Japan

^4^Tropical Biosphere Research Center, University of the Ryukyus, 1 Senbaru, Nishihara, Okinawa, 903-0213, Japan

^5^School of Environmental and Life Sciences, The University of Newcastle, Callaghan, NSW, 2308, Australia

^6^School of Biology, Faculty of Biological Sciences, University of Leeds, Leeds, LS2 9JT, United Kingdom

^7^School of Biological Sciences, Victoria University of Wellington, PO Box 600, Wellington, 6140, New Zealand

^8^Department of Biological Sciences, Macquarie University, North Ryde, NSW, 2113, Australia

^9^Environmental Protection Authority, 215 Lambton Quay, Wellington, 6011, New Zealand

**Fig S1**. **Principal Coordinate Analysis (PCO) comparing presence/absence of eukaryotic families detected based on 18S rRNA sequences amplified from sediment and seawater samples collected at 14 coastal sites in Okinawa, Japan**. The relationship of eukaryotic community assemblages identified in each sample using a Jaccard index for the factor “method” is shown, the proportion of variation explained is outlined on each axis, and the different methods are indicated by colours in the legend.


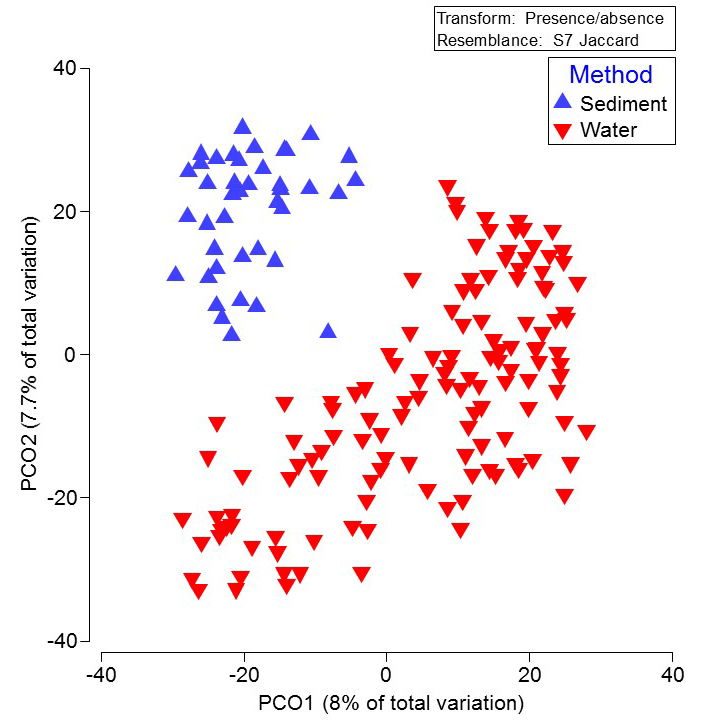

Supplement: Supplementary file 1 — Supplementary information. [file 41598_2020_64858_MOESM1_ESM.docx]
