# Supplementary material for: Environmental DNA can act as a biodiversity barometer of anthropogenic pressures in coastal ecosystems
Source: Sci Rep. 2020 May 20;10:8365. doi: 10.1038/s41598-020-64858-9 (PMC7239923; doi:10.1038/s41598-020-64858-9)

**Environmental DNA can act as a biodiversity barometer of anthropogenic pressures in coastal ecosystems**

**Joseph D. DiBattista^1,2*^, James D. Reimer^3,4^, Michael Stat^1,5^, Giovanni D. Masucci^3^, Piera Biondi^3^, Maarten De Brauwer^1,6^, Shaun P. Wilkinson^7^, Anthony A. Chariton^8^, Michael Bunce^1,9^**

Author affiliations:

^1^Trace and Environmental DNA (TrEnD) Laboratory, School of Molecular and Life Sciences, Curtin University, Perth, WA, 6102, Australia

^2^Australian Museum Research Institute, Australian Museum, 1 William St, Sydney, NSW, 2010, Australia

^3^Molecular Invertebrate and Systematics Ecology Laboratory, Graduate School of Engineering and Science, University of the Ryukyus, 1 Senbaru, Nishihara, Okinawa, 903-0213, Japan

^4^Tropical Biosphere Research Center, University of the Ryukyus, 1 Senbaru, Nishihara, Okinawa, 903-0213, Japan

^5^School of Environmental and Life Sciences, The University of Newcastle, Callaghan, NSW, 2308, Australia

^6^School of Biology, Faculty of Biological Sciences, University of Leeds, Leeds, LS2 9JT, United Kingdom

^7^School of Biological Sciences, Victoria University of Wellington, PO Box 600, Wellington, 6140, New Zealand

^8^Department of Biological Sciences, Macquarie University, North Ryde, NSW, 2113, Australia

^9^Environmental Protection Authority, 215 Lambton Quay, Wellington, 6011, New Zealand

**Fig. S2.** **Principal Component Analysis (PCO) of the presence/absence of eukaryotic families detected based on 18S rRNA sequences from sediment (A) and seawater (B) collected at 14 sites off the coast of Okinawa, Japan**. The relationship of eukaryotic community assemblages identified in each sample using a Jaccard index with labels above indicating the year that the samples were collected. The proportion of variation explained is outlined on each axis and the different sites are indicated by symbols in the legend.


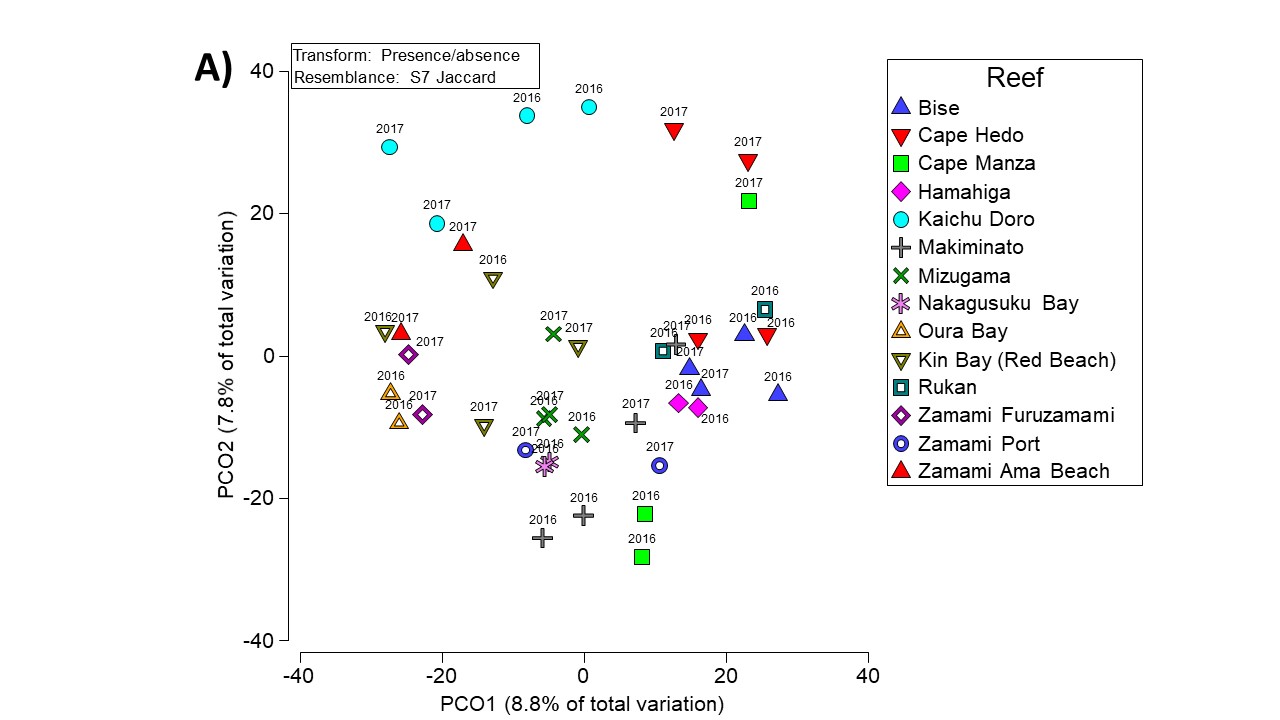


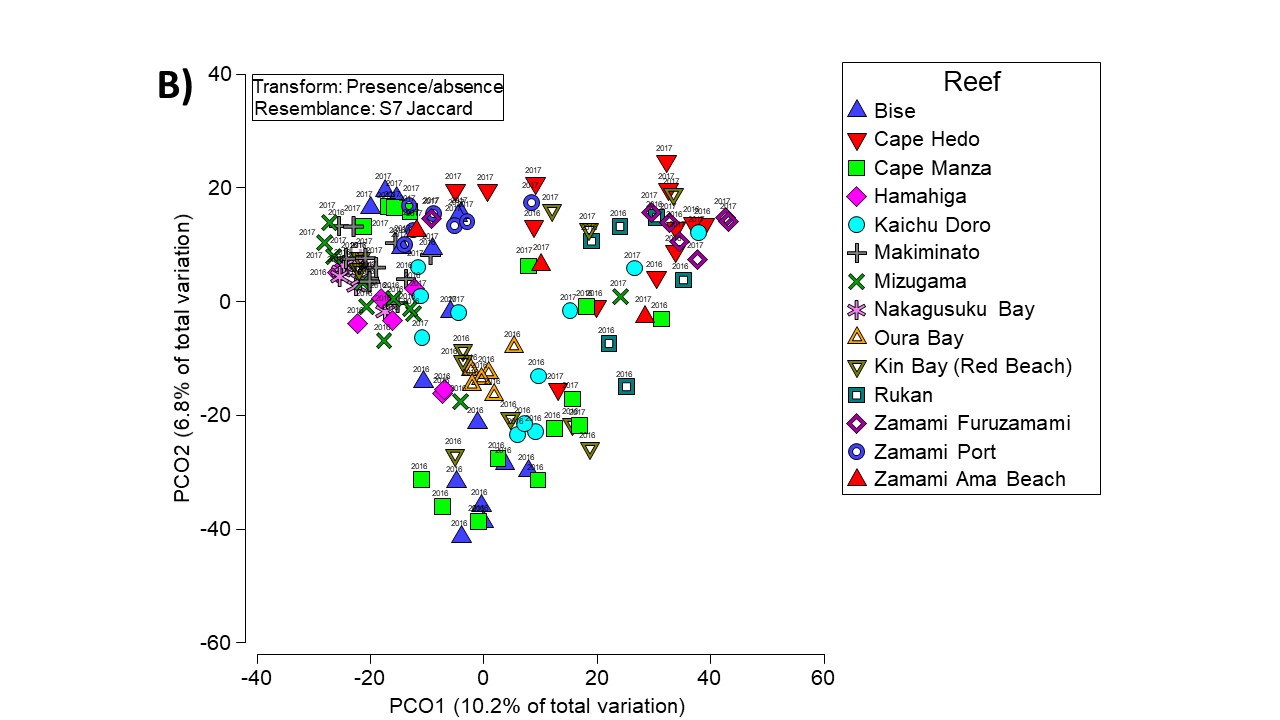

Supplement: Supplementary file 2 — Supplementary information2. [file 41598_2020_64858_MOESM2_ESM.docx]
