# Supplementary material for: Environmental DNA can act as a biodiversity barometer of anthropogenic pressures in coastal ecosystems
Source: Sci Rep. 2020 May 20;10:8365. doi: 10.1038/s41598-020-64858-9 (PMC7239923; doi:10.1038/s41598-020-64858-9)
Supplement: Supplementary file 4 — Supplementary information4. [file 41598_2020_64858_MOESM4_ESM.docx]

**Environmental DNA can act as a biodiversity barometer of anthropogenic pressures in coastal ecosystems**

**Joseph D. DiBattista^1,2*^, James D. Reimer^3,4^, Michael Stat^1,5^, Giovanni D. Masucci^3^, Piera Biondi^3^, Maarten De Brauwer^1,6^, Shaun P. Wilkinson^7^, Anthony A. Chariton^8^, Michael Bunce^1,9^**

Author affiliations:

^1^Trace and Environmental DNA (TrEnD) Laboratory, School of Molecular and Life Sciences, Curtin University, Perth, WA, 6102, Australia

^2^Australian Museum Research Institute, Australian Museum, 1 William St, Sydney, NSW, 2010, Australia

^3^Molecular Invertebrate and Systematics Ecology Laboratory, Graduate School of Engineering and Science, University of the Ryukyus, 1 Senbaru, Nishihara, Okinawa, 903-0213, Japan

^4^Tropical Biosphere Research Center, University of the Ryukyus, 1 Senbaru, Nishihara, Okinawa, 903-0213, Japan

^5^School of Environmental and Life Sciences, The University of Newcastle, Callaghan, NSW, 2308, Australia

^6^School of Biology, Faculty of Biological Sciences, University of Leeds, Leeds, LS2 9JT, United Kingdom

^7^School of Biological Sciences, Victoria University of Wellington, PO Box 600, Wellington, 6140, New Zealand

^8^Department of Biological Sciences, Macquarie University, North Ryde, NSW, 2113, Australia

^9^Environmental Protection Authority, 215 Lambton Quay, Wellington, 6011, New Zealand

**Table S1**. **18S rRNA and ITS2 sequence information per replicate for sediment and seawater samples collected at 14 sites off the coast of Okinawa, Japan**. “Assigned unique reads” refers to the number of unique reads assigned at the family-level in MEGAN v 5.11.3 (MEGAN run parameters outlined in the Materials and Methods); “total unique reads” refers to the number of dereplicated sequences after QF, including sub-sampling 20,000 reads from each 18S rRNA replicate. Due to the lower amplification success of the ITS2 marker, replicates within sites were merged and subsampled to 20,000 and 4,000 reads prior to dereplication for sediment and seawater samples, respectively. Zero reads indicate no amplification, whereas “n/a” indicates that the sample was not processed.

| Sample_ID | Location | Year | Latitude | Longitude | Number of 18S reads | Post-QF 18S *N* > 2  assigned unique reads (total unique reads) | Number of ITS2 reads | Post-QF ITS2, *N* > 2  assigned unique reads (total unique reads) |
| --- | --- | --- | --- | --- | --- | --- | --- | --- |
| Sediment |  |  |  |  |  |  |  |  |
| AWFS_F16_0415 | Bise | 2016 | 26.71195 | 127.88122 | 64,625 | 144 (718) | 32,005 | 339 (553) |
| AWFS_F16_0416 | Bise | 2016 | 26.71195 | 127.88122 | 71,221 | 255 (652) | 52,561 | 322 (924) |
| SED92 | Bise | 2017 | 26.71195 | 127.88122 | 110,442 | 281 (850) | n/a | n/a |
| SED93 | Bise | 2017 | 26.71195 | 127.88122 | 72,059 | 331 (918) | n/a | n/a |
| AWFS_F16_0427 | Cape Hedo | 2016 | 26.87228 | 128.26652 | 54,725 | 597 (805) | 177,091 | 154 (2,049) |
| AWFS_F16_0428 | Cape Hedo | 2016 | 26.87228 | 128.26652 | 101,967 | 564 (677) | 46,679 | 172 (1,106) |
| SED120 | Cape Hedo | 2017 | 26.87228 | 128.26652 | 90,789 | 253 (989) | n/a | n/a |
| SED121 | Cape Hedo | 2017 | 26.87228 | 128.26652 | 202,696 | 602 (669) | n/a | n/a |
| AWFS_F16_0413 | Cape Manza | 2016 | 26.51377 | 127.86374 | 73,110 | 208 (747) | 107,159 | 1,287 (1,420) |
| AWFS_F16_0414 | Cape Manza | 2016 | 26.51377 | 127.86374 | 70,404 | 259 (774) | 2,461 | 3 (84) |
| SED116 | Cape Manza | 2017 | 26.51377 | 127.86374 | 16,469 | 0 (0) | n/a | n/a |
| SED117 | Cape Manza | 2017 | 26.51377 | 127.86374 | 28,694 | 473 (642) | n/a | n/a |
| AWFS_F16_0411 | Hamahiga | 2016 | 26.31184 | 127.96133 | 73,931 | 89 (743) | 19,557 | 1 (486) |
| AWFS_F16_0412 | Hamahiga | 2016 | 26.31184 | 127.96133 | 50,097 | 172 (750) | 30,137 | 0 (761) |
| AWFS_F16_0435 | Kaichu Doro S1 | 2016 | 26.33086 | 127.90625 | 85,468 | 400 (440) | 197,200 | 3 (3,138) |
| AWFS_F16_0436 | Kaichu Doro S1 | 2016 | 26.33086 | 127.90625 | 128,966 | 547 (570) | 275,577 | 20 (1,743) |
| SED88 | Kaichu Doro S1 | 2017 | 26.33086 | 127.90625 | 122,565 | 613 (906) | n/a | n/a |
| SED89 | Kaichu Doro S1 | 2017 | 26.33086 | 127.90625 | 126,216 | 474 (960) | n/a | n/a |
| AWFS_F16_0425 | Makiminato | 2016 | 26.27493 | 127.71393 | 53,402 | 352 (712) | 40,743 | 165 (1,357) |
| AWFS_F16_0426 | Makiminato | 2016 | 26.27493 | 127.71393 | 47,037 | 447 (764) | 91,503 | 149 (2,025) |
| SED96 | Makiminato | 2017 | 26.27493 | 127.71393 | 38,892 | 611 (911) | n/a | n/a |
| SED97 | Makiminato | 2017 | 26.27493 | 127.71393 | 139,059 | 378 (560) | n/a | n/a |
| AWFS_F16_0409 | Mizugama | 2016 | 26.36013 | 127.73919 | 24,121 | 306 (489) | 31,584 | 47 (681) |
| AWFS_F16_0410 | Mizugama | 2016 | 26.36013 | 127.73919 | 64,469 | 484 (798) | 34,749 | 130 (848) |
| SED100 | Mizugama | 2017 | 26.36013 | 127.73919 | 36,423 | 379 (855) | n/a | n/a |
| SED101 | Mizugama | 2017 | 26.36013 | 127.73919 | 35,938 | 421 (870) | n/a | n/a |
| AWFS_F16_0423 | Nakagusuku Bay | 2016 | 26.21548 | 127.77611 | 92,774 | 310 (466) | 38,688 | 316 (1,126) |
| AWFS_F16_0424 | Nakagusuku Bay | 2016 | 26.21548 | 127.77611 | 33,399 | 512 (768) | 46,078 | 108 (1,083) |
| AWFS_F16_0421 | Oura Bay | 2016 | 26.54635 | 128.05022 | 84,094 | 545 (827) | 24,091 | 4 (766) |
| AWFS_F16_0422 | Oura Bay | 2016 | 26.54635 | 128.05022 | 106,181 | 639 (746) | 77,688 | 0 (1,636) |
| AWFS_F16_0419 | Kin Bay (Red Beach) | 2016 | 26.44700 | 127.91272 | 44,953 | 506 (934) | 102,303 | 1 (1,740) |
| AWFS_F16_0420 | Kin Bay (Red Beach) | 2016 | 26.44700 | 127.91272 | 115,056 | 241 (776) | 73,152 | 3 (1,199) |
| SED84 | Kin Bay (Red Beach) | 2017 | 26.44700 | 127.91272 | 53,356 | 705 (1,012) | n/a | n/a |
| SED85 | Kin Bay (Red Beach) | 2017 | 26.44700 | 127.91272 | 73,459 | 662 (989) | n/a | n/a |
| AWFS_F16_0437 | Rukan | 2016 | 26.09961 | 127.53962 | 28,112 | 626 (800) | 0 | 0 (0) |
| AWFS_F16_0438 | Rukan | 2016 | 26.09961 | 127.53962 | 93,017 | 94 (613) | 0 | 0 (0) |
| SED104 | Zamami (Ama Beach) | 2017 | 26.22670 | 127.29239 | 42,984 | 384 (781) | n/a | n/a |
| SED105 | Zamami (Ama Beach) | 2017 | 26.22670 | 127.29239 | 31,449 | 368 (791) | n/a | n/a |
| SED108 | Zamami (Port) | 2017 | 26.22766 | 127.30138 | 62,186 | 298 (456) | n/a | n/a |
| SED109 | Zamami (Port) | 2017 | 26.22766 | 127.30138 | 41,213 | 438 (737) | n/a | n/a |
| SED112 | Zamami (Furuzamami) | 2017 | 26.22261 | 127.30898 | 37,384 | 536 (1,181) | n/a | n/a |
| SED113 | Zamami (Furuzamami) | 2017 | 26.22261 | 127.30898 | 21,786 | 548 (1,162) | n/a | n/a |
|  |  |  |  |  |  |  |  |  |
| Water |  |  |  |  |  |  |  |  |
| OKI25 | Bise | 2016 | 26.71195 | 127.88122 | 37,613 | 561 (618) | 15,952 | 7 (335) |
| OKI26 | Bise | 2016 | 26.71195 | 127.88122 | 53,165 | 610 (654) | 21,960 | 76 (526) |
| OKI27 | Bise | 2016 | 26.71195 | 127.88122 | 115,360 | 322 (550) | 12,837 | 78 (346) |
| OKI28 | Bise | 2016 | 26.71195 | 127.88122 | 62,178 | 530 (599) | 22,655 | 9 (417) |
| OKI29 | Bise | 2016 | 26.71195 | 127.88122 | 86,214 | 566 (697) | 48,320 | 318 (930) |
| OKI30 | Bise | 2016 | 26.71195 | 127.88122 | 74,066 | 594 (614) | 1,001 | 0 (35) |
| OKI31 | Bise | 2016 | 26.71195 | 127.88122 | 57,664 | 515 (607) | 13,180 | 149 (372) |
| OKI32 | Bise | 2016 | 26.71195 | 127.88122 | 80,949 | 243 (733) | 36,382 | 390 (835) |
| OKI101 | Bise | 2017 | 26.71195 | 127.88122 | 66,530 | 473 (907) | n/a | n/a |
| OKI102 | Bise | 2017 | 26.71195 | 127.88122 | 103,346 | 184 (770) | n/a | n/a |
| OKI103 | Bise | 2017 | 26.71195 | 127.88122 | 87,687 | 310 (793) | n/a | n/a |
| OKI104 | Bise | 2017 | 26.71195 | 127.88122 | 71,720 | 361 (817) | n/a | n/a |
| OKI105 | Bise | 2017 | 26.71195 | 127.88122 | 109,085 | 584 (662) | n/a | n/a |
| OKI106 | Bise | 2017 | 26.71195 | 127.88122 | 54,425 | 410 (963) | n/a | n/a |
| OKI107 | Bise | 2017 | 26.71195 | 127.88122 | 72,721 | 467 (822) | n/a | n/a |
| OKI108 | Bise | 2017 | 26.71195 | 127.88122 | 89,469 | 421 (723) | n/a | n/a |
| OKI65 | Cape Hedo | 2016 | 26.87228 | 128.26652 | 110,209 | 27 (597) | 0 | 0 (0) |
| OKI66 | Cape Hedo | 2016 | 26.87228 | 128.26652 | 80,488 | 359 (624) | 0 | 0 (0) |
| OKI67 | Cape Hedo | 2016 | 26.87228 | 128.26652 | 150,065 | 196 (469) | 0 | 0 (0) |
| OKI68 | Cape Hedo | 2016 | 26.87228 | 128.26652 | 79,251 | 570 (637) | 0 | 0 (0) |
| OKI69 | Cape Hedo | 2016 | 26.87228 | 128.26652 | 172,346 | 209 (856) | 1,545 | 25 (60) |
| OKI70 | Cape Hedo | 2016 | 26.87228 | 128.26652 | 12,927 | 0 (0) | 0 | 0 (0) |
| OKI71 | Cape Hedo | 2016 | 26.87228 | 128.26652 | 45,458 | 172 (495) | 2,988 | 19 (89) |
| OKI72 | Cape Hedo | 2016 | 26.87228 | 128.26652 | 133,681 | 308 (596) | 1,638 | 11 (50) |
| OKI157 | Cape Hedo | 2017 | 26.87228 | 128.26652 | 127,742 | 318 (701) | n/a | n/a |
| OKI158 | Cape Hedo | 2017 | 26.87228 | 128.26652 | 16,889 | 0 (0) | n/a | n/a |
| OKI159 | Cape Hedo | 2017 | 26.87228 | 128.26652 | 190,035 | 299 (610) | n/a | n/a |
| OKI160 | Cape Hedo | 2017 | 26.87228 | 128.26652 | 31,355 | 254 (460) | n/a | n/a |
| OKI161 | Cape Hedo | 2017 | 26.87228 | 128.26652 | 116,413 | 440 (900) | n/a | n/a |
| OKI162 | Cape Hedo | 2017 | 26.87228 | 128.26652 | 17,655 | 0 (0) | n/a | n/a |
| OKI163 | Cape Hedo | 2017 | 26.87228 | 128.26652 | 106,208 | 167 (521) | n/a | n/a |
| OKI164 | Cape Hedo | 2017 | 26.87228 | 128.26652 | 159,095 | 154 (601) | n/a | n/a |
| OKI17 | Cape Manza | 2016 | 26.51377 | 127.86374 | 147,466 | 500 (668) | 67,028 | 325 (1,164) |
| OKI18 | Cape Manza | 2016 | 26.51377 | 127.86374 | 56,588 | 79 (640) | 13,011 | 257 (290) |
| OKI19 | Cape Manza | 2016 | 26.51377 | 127.86374 | 59,777 | 296 (559) | 1,854 | 10 (48) |
| OKI20 | Cape Manza | 2016 | 26.51377 | 127.86374 | 81,871 | 93 (585) | 22,285 | 314 (321) |
| OKI21 | Cape Manza | 2016 | 26.51377 | 127.86374 | 75,819 | 467 (703) | 6,793 | 112 (181) |
| OKI22 | Cape Manza | 2016 | 26.51377 | 127.86374 | 40,049 | 116 (649) | 5,450 | 92 (116) |
| OKI23 | Cape Manza | 2016 | 26.51377 | 127.86374 | 74,951 | 120 (621) | 36,396 | 464 (628) |
| OKI24 | Cape Manza | 2016 | 26.51377 | 127.86374 | 71,780 | 46 (513) | 338 | 5 (8) |
| OKI149 | Cape Manza | 2017 | 26.51377 | 127.86374 | 20,638 | 415 (763) | n/a | n/a |
| OKI150 | Cape Manza | 2017 | 26.51377 | 127.86374 | 39,624 | 27 (630) | n/a | n/a |
| OKI151 | Cape Manza | 2017 | 26.51377 | 127.86374 | 21,020 | 408 (819) | n/a | n/a |
| OKI152 | Cape Manza | 2017 | 26.51377 | 127.86374 | 43,465 | 81 (494) | n/a | n/a |
| OKI153 | Cape Manza | 2017 | 26.51377 | 127.86374 | 27,336 | 556 (665) | n/a | n/a |
| OKI154 | Cape Manza | 2017 | 26.51377 | 127.86374 | 22,584 | 392 (715) | n/a | n/a |
| OKI155 | Cape Manza | 2017 | 26.51377 | 127.86374 | 108,686 | 426 (902) | n/a | n/a |
| OKI156 | Cape Manza | 2017 | 26.51377 | 127.86374 | 17,996 | 0 (0) | n/a | n/a |
| OKI9 | Hamahiga | 2016 | 26.31184 | 127.96133 | 138,079 | 466 (695) | 146,496 | 315 (2,027) |
| OKI10 | Hamahiga | 2016 | 26.31184 | 127.96133 | 80,636 | 380 (651) | 32,599 | 149 (640) |
| OKI11 | Hamahiga | 2016 | 26.31184 | 127.96133 | 89,818 | 268 (643) | 25,690 | 18 (495) |
| OKI12 | Hamahiga | 2016 | 26.31184 | 127.96133 | 182,621 | 509 (787) | 32,494 | 33 (508) |
| OKI13 | Hamahiga | 2016 | 26.31184 | 127.96133 | 0 | 0 (0) | 0 | 0 (0) |
| OKI14 | Hamahiga | 2016 | 26.31184 | 127.96133 | 91,672 | 105 (681) | 168,659 | 36 (1,600) |
| OKI15 | Hamahiga | 2016 | 26.31184 | 127.96133 | 58,786 | 249 (540) | 29,411 | 50 (714) |
| OKI16 | Hamahiga | 2016 | 26.31184 | 127.96133 | 67,127 | 602 (781) | 303,878 | 366 (2,938) |
| OKI73 | Kaichu Doro S1 | 2016 | 26.33086 | 127.90625 | 74,193 | 610 (633) | 4,862 | 127 (164) |
| OKI74 | Kaichu Doro S1 | 2016 | 26.33086 | 127.90625 | 48,779 | 588 (616) | 688 | 13 (25) |
| OKI75 | Kaichu Doro S1 | 2016 | 26.33086 | 127.90625 | 70,626 | 527 (550) | 939 | 11 (35) |
| OKI76 | Kaichu Doro S1 | 2016 | 26.33086 | 127.90625 | 66,974 | 603 (642) | 18,158 | 7 (425) |
| OKI93 | Kaichu Doro S1 | 2017 | 26.33086 | 127.90625 | 223,002 | 539 (710) | n/a | n/a |
| OKI94 | Kaichu Doro S1 | 2017 | 26.33086 | 127.90625 | 75,608 | 530 (671) | n/a | n/a |
| OKI95 | Kaichu Doro S1 | 2017 | 26.33086 | 127.90625 | 114,045 | 527 (741) | n/a | n/a |
| OKI96 | Kaichu Doro S1 | 2017 | 26.33086 | 127.90625 | 0 | 0 (0) | n/a | n/a |
| OKI97 | Kaichu Doro S1 | 2017 | 26.33086 | 127.90625 | 129,903 | 547 (752) | n/a | n/a |
| OKI98 | Kaichu Doro S1 | 2017 | 26.33086 | 127.90625 | 61,418 | 306 (604) | n/a | n/a |
| OKI99 | Kaichu Doro S1 | 2017 | 26.33086 | 127.90625 | 57,925 | 494 (693) | n/a | n/a |
| OKI100 | Kaichu Doro S1 | 2017 | 26.33086 | 127.90625 | 89,232 | 520 (568) | n/a | n/a |
| OKI57 | Makiminato | 2016 | 26.27493 | 127.71393 | 0 | 0 (0) | 1,551 | 0 (53) |
| OKI58 | Makiminato | 2016 | 26.27493 | 127.71393 | 45,773 | 296 (509) | 984 | 1 (30) |
| OKI59 | Makiminato | 2016 | 26.27493 | 127.71393 | 120,157 | 444 (740) | 12,272 | 89 (265) |
| OKI60 | Makiminato | 2016 | 26.27493 | 127.71393 | 0 | 0 (0) | 0 | 0 (0) |
| OKI61 | Makiminato | 2016 | 26.27493 | 127.71393 | 168,055 | 459 (843) | 1,022 | 13 (48) |
| OKI62 | Makiminato | 2016 | 26.27493 | 127.71393 | 48,201 | 468 (657) | 854 | 8 (26) |
| OKI63 | Makiminato | 2016 | 26.27493 | 127.71393 | 32,535 | 445 (662) | 857 | 13 (21) |
| OKI64 | Makiminato | 2016 | 26.27493 | 127.71393 | 83,618 | 519 (717) | 12,283 | 100 (233) |
| OKI109 | Makiminato | 2017 | 26.27493 | 127.71393 | 26,960 | 466 (661) | n/a | n/a |
| OKI110 | Makiminato | 2017 | 26.27493 | 127.71393 | 218,654 | 418 (510) | n/a | n/a |
| OKI111 | Makiminato | 2017 | 26.27493 | 127.71393 | 9,177 | 0 (0) | n/a | n/a |
| OKI112 | Makiminato | 2017 | 26.27493 | 127.71393 | 22,678 | 417 (649) | n/a | n/a |
| OKI113 | Makiminato | 2017 | 26.27493 | 127.71393 | 45,101 | 485 (670) | n/a | n/a |
| OKI114 | Makiminato | 2017 | 26.27493 | 127.71393 | 171,551 | 409 (545) | n/a | n/a |
| OKI115 | Makiminato | 2017 | 26.27493 | 127.71393 | 26,754 | 570 (845) | n/a | n/a |
| OKI116 | Makiminato | 2017 | 26.27493 | 127.71393 | 29,012 | 544 (722) | n/a | n/a |
| OKI1 | Mizugama | 2016 | 26.36013 | 127.73919 | 66,169 | 633 (793) | 244,834 | 2,635 (4,247) |
| OKI2 | Mizugama | 2016 | 26.36013 | 127.73919 | 179,600 | 360 (477) | 60,530 | 1,100 (1,312) |
| OKI3 | Mizugama | 2016 | 26.36013 | 127.73919 | 117,339 | 460 (554) | 36,148 | 641 (642) |
| OKI4 | Mizugama | 2016 | 26.36013 | 127.73919 | 57,630 | 607 (851) | 179,498 | 1,780 (3,826) |
| OKI5 | Mizugama | 2016 | 26.36013 | 127.73919 | 71,149 | 253 (678) | 23,194 | 505 (531) |
| OKI6 | Mizugama | 2016 | 26.36013 | 127.73919 | 30,515 | 364 (502) | 152,219 | 1,828 (3,307) |
| OKI7 | Mizugama | 2016 | 26.36013 | 127.73919 | 0 | 0 (0) | 130,035 | 2,193 (2,766) |
| OKI8 | Mizugama | 2016 | 26.36013 | 127.73919 | 0 | 0 (0) | 95,497 | 1,103 (2,007) |
| OKI117 | Mizugama | 2017 | 26.36013 | 127.73919 | 228,929 | 551 (680) | n/a | n/a |
| OKI118 | Mizugama | 2017 | 26.36013 | 127.73919 | 27,793 | 397 (760) | n/a | n/a |
| OKI119 | Mizugama | 2017 | 26.36013 | 127.73919 | 28,445 | 563 (694) | n/a | n/a |
| OKI120 | Mizugama | 2017 | 26.36013 | 127.73919 | 257,894 | 465 (540) | n/a | n/a |
| OKI121 | Mizugama | 2017 | 26.36013 | 127.73919 | 11,770 | 0 (0) | n/a | n/a |
| OKI122 | Mizugama | 2017 | 26.36013 | 127.73919 | 16,084 | 0 (0) | n/a | n/a |
| OKI123 | Mizugama | 2017 | 26.36013 | 127.73919 | 18,588 | 0 (0) | n/a | n/a |
| OKI124 | Mizugama | 2017 | 26.36013 | 127.73919 | 185,996 | 381 (696) | n/a | n/a |
| OKI49 | Nakagusuku Bay | 2016 | 26.21548 | 127.77611 | 75,412 | 254 (751) | 2,811 | 13 (70) |
| OKI50 | Nakagusuku Bay | 2016 | 26.21548 | 127.77611 | 15,670 | 0 (0) | 4,945 | 117 (161) |
| OKI51 | Nakagusuku Bay | 2016 | 26.21548 | 127.77611 | 60,234 | 496 (680) | 2,080 | 8 (58) |
| OKI52 | Nakagusuku Bay | 2016 | 26.21548 | 127.77611 | 51,854 | 560 (807) | 10,835 | 76 (254) |
| OKI53 | Nakagusuku Bay | 2016 | 26.21548 | 127.77611 | 64,945 | 654 (762) | 4,554 | 83 (125) |
| OKI54 | Nakagusuku Bay | 2016 | 26.21548 | 127.77611 | 88,400 | 530 (736) | 3,573 | 40 (93) |
| OKI55 | Nakagusuku Bay | 2016 | 26.21548 | 127.77611 | 184,799 | 558 (763) | 6,156 | 50 (154) |
| OKI56 | Nakagusuku Bay | 2016 | 26.21548 | 127.77611 | 134,823 | 384 (635) | 2,818 | 46 (64) |
| OKI41 | Oura Bay | 2016 | 26.54635 | 128.05022 | 48,190 | 316 (634) | 7,756 | 198 (244) |
| OKI42 | Oura Bay | 2016 | 26.54635 | 128.05022 | 65,551 | 644 (711) | 14,704 | 1 (318) |
| OKI43 | Oura Bay | 2016 | 26.54635 | 128.05022 | 89,770 | 581 (656) | 3,957 | 110 (128) |
| OKI44 | Oura Bay | 2016 | 26.54635 | 128.05022 | 53,282 | 450 (508) | 13,254 | 0 (396) |
| OKI45 | Oura Bay | 2016 | 26.54635 | 128.05022 | 11,165 | 0 (0) | 24,797 | 5 (390) |
| OKI46 | Oura Bay | 2016 | 26.54635 | 128.05022 | 18,404 | 0 (0) | 12,147 | 0 (276) |
| OKI47 | Oura Bay | 2016 | 26.54635 | 128.05022 | 35,749 | 685 (747) | 3,124 | 2 (134) |
| OKI48 | Oura Bay | 2016 | 26.54635 | 128.05022 | 38,084 | 528 (612) | 2,447 | 8 (66) |
| OKI33 | Kin Bay (Red Beach) | 2016 | 26.44700 | 127.91272 | 8,955 | 0 (0) | 20,466 | 317 (567) |
| OKI34 | Kin Bay (Red Beach) | 2016 | 26.44700 | 127.91272 | 1,397 | 0 (0) | 16,291 | 314 (452) |
| OKI35 | Kin Bay (Red Beach) | 2016 | 26.44700 | 127.91272 | 26,040 | 464 (549) | 15,370 | 90 (421) |
| OKI36 | Kin Bay (Red Beach) | 2016 | 26.44700 | 127.91272 | 31,846 | 490 (674) | 22,563 | 328 (529) |
| OKI37 | Kin Bay (Red Beach) | 2016 | 26.44700 | 127.91272 | 67,034 | 578 (623) | 3,954 | 130 (139) |
| OKI38 | Kin Bay (Red Beach) | 2016 | 26.44700 | 127.91272 | 20,559 | 184 (643) | 626 | 0 (3) |
| OKI39 | Kin Bay (Red Beach) | 2016 | 26.44700 | 127.91272 | 61,571 | 353 (654) | 0 | 0 (0) |
| OKI40 | Kin Bay (Red Beach) | 2016 | 26.44700 | 127.91272 | 29,404 | 532 (686) | 3,893 | 50 (82) |
| OKI85 | Kin Bay (Red Beach) | 2017 | 26.44700 | 127.91272 | 62,943 | 276 (597) | n/a | n/a |
| OKI86 | Kin Bay (Red Beach) | 2017 | 26.44700 | 127.91272 | 53,886 | 367 (704) | n/a | n/a |
| OKI87 | Kin Bay (Red Beach) | 2017 | 26.44700 | 127.91272 | 90,415 | 359 (632) | n/a | n/a |
| OKI88 | Kin Bay (Red Beach) | 2017 | 26.44700 | 127.91272 | 78,940 | 650 (892) | n/a | n/a |
| OKI89 | Kin Bay (Red Beach) | 2017 | 26.44700 | 127.91272 | 0 | 0 (0) | n/a | n/a |
| OKI90 | Kin Bay (Red Beach) | 2017 | 26.44700 | 127.91272 | 0 | 0 (0) | n/a | n/a |
| OKI91 | Kin Bay (Red Beach) | 2017 | 26.44700 | 127.91272 | 86,621 | 548 (717) | n/a | n/a |
| OKI92 | Kin Bay (Red Beach) | 2017 | 26.44700 | 127.91272 | 0 | 0 (0) | n/a | n/a |
| OKI77 | Rukan | 2016 | 26.09961 | 127.53962 | 22,759 | 185 (758) | 270 | 6 (14) |
| OKI78 | Rukan | 2016 | 26.09961 | 127.53962 | 65,532 | 563 (704) | 0 | 0 (0) |
| OKI79 | Rukan | 2016 | 26.09961 | 127.53962 | 127,956 | 175 (576) | 4,685 | 1 (153) |
| OKI80 | Rukan | 2016 | 26.09961 | 127.53962 | 6,200 | 0 (0) | 0 | 0 (0) |
| OKI81 | Rukan | 2016 | 26.09961 | 127.53962 | 25,906 | 500 (659) | 0 | 0 (0) |
| OKI82 | Rukan | 2016 | 26.09961 | 127.53962 | 18,048 | 0 (0) | 0 | 0 (0) |
| OKI83 | Rukan | 2016 | 26.09961 | 127.53962 | 25,217 | 248 (498) | 0 | 0 (0) |
| OKI84 | Rukan | 2016 | 26.09961 | 127.53962 | 36,860 | 36 (506) | 0 | 0 (0) |
| OKI125 | Zamami (Ama Beach) | 2017 | 26.22670 | 127.29239 | 19,980 | 0 (0) | n/a | n/a |
| OKI126 | Zamami (Ama Beach) | 2017 | 26.22670 | 127.29239 | 17,532 | 0 (0) | n/a | n/a |
| OKI127 | Zamami (Ama Beach) | 2017 | 26.22670 | 127.29239 | 15,591 | 0 (0) | n/a | n/a |
| OKI128 | Zamami (Ama Beach) | 2017 | 26.22670 | 127.29239 | 31,377 | 39 (572) | n/a | n/a |
| OKI129 | Zamami (Ama Beach) | 2017 | 26.22670 | 127.29239 | 109,390 | 515 (1,025) | n/a | n/a |
| OKI130 | Zamami (Ama Beach) | 2017 | 26.22670 | 127.29239 | 16,494 | 0 (0) | n/a | n/a |
| OKI131 | Zamami (Ama Beach) | 2017 | 26.22670 | 127.29239 | 0 | 0 (0) | n/a | n/a |
| OKI132 | Zamami (Ama Beach) | 2017 | 26.22670 | 127.29239 | 30,312 | 147 (710) | n/a | n/a |
| OKI133 | Zamami (Port) | 2017 | 26.22766 | 127.30138 | 25,631 | 170 (500) | n/a | n/a |
| OKI134 | Zamami (Port) | 2017 | 26.22766 | 127.30138 | 22,219 | 360 (651) | n/a | n/a |
| OKI135 | Zamami (Port) | 2017 | 26.22766 | 127.30138 | 243,025 | 232 (531) | n/a | n/a |
| OKI136 | Zamami (Port) | 2017 | 26.22766 | 127.30138 | 32,844 | 312 (708) | n/a | n/a |
| OKI137 | Zamami (Port) | 2017 | 26.22766 | 127.30138 | 14,236 | 0 (0) | n/a | n/a |
| OKI138 | Zamami (Port) | 2017 | 26.22766 | 127.30138 | 28,717 | 369 (705) | n/a | n/a |
| OKI139 | Zamami (Port) | 2017 | 26.22766 | 127.30138 | 31,569 | 313 (586) | n/a | n/a |
| OKI140 | Zamami (Port) | 2017 | 26.22766 | 127.30138 | 41,417 | 264 (609) | n/a | n/a |
| OKI141 | Zamami (Furuzamami) | 2017 | 26.22261 | 127.30898 | 36,012 | 216 (705) | n/a | n/a |
| OKI142 | Zamami (Furuzamami) | 2017 | 26.22261 | 127.30898 | 34,170 | 82 (552) | n/a | n/a |
| OKI143 | Zamami (Furuzamami) | 2017 | 26.22261 | 127.30898 | 32,007 | 141 (458) | n/a | n/a |
| OKI144 | Zamami (Furuzamami) | 2017 | 26.22261 | 127.30898 | 17,651 | 0 (0) | n/a | n/a |
| OKI145 | Zamami (Furuzamami) | 2017 | 26.22261 | 127.30898 | 30,638 | 317 (703) | n/a | n/a |
| OKI146 | Zamami (Furuzamami) | 2017 | 26.22261 | 127.30898 | 27,916 | 93 (535) | n/a | n/a |
| OKI147 | Zamami (Furuzamami) | 2017 | 26.22261 | 127.30898 | 299,631 | 96 (574) | n/a | n/a |
| OKI148 | Zamami (Furuzamami) | 2017 | 26.22261 | 127.30898 | 167,500 | 403 (614) | n/a | n/a |
