# Supplementary material for: Environmental DNA can act as a biodiversity barometer of anthropogenic pressures in coastal ecosystems
Source: Sci Rep. 2020 May 20;10:8365. doi: 10.1038/s41598-020-64858-9 (PMC7239923; doi:10.1038/s41598-020-64858-9)
Supplement: Supplementary file 5 — Supplementary information5. [file 41598_2020_64858_MOESM5_ESM.docx]

**Environmental DNA can act as a biodiversity barometer of anthropogenic pressures in coastal ecosystems**

**Joseph D. DiBattista^1,2*^, James D. Reimer^3,4^, Michael Stat^1,5^, Giovanni D. Masucci^3^, Piera Biondi^3^, Maarten De Brauwer^1,6^, Shaun P. Wilkinson^7^, Anthony A. Chariton^8^, Michael Bunce^1,9^**

Author affiliations:

^1^Trace and Environmental DNA (TrEnD) Laboratory, School of Molecular and Life Sciences, Curtin University, Perth, WA, 6102, Australia

^2^Australian Museum Research Institute, Australian Museum, 1 William St, Sydney, NSW, 2010, Australia

^3^Molecular Invertebrate and Systematics Ecology Laboratory, Graduate School of Engineering and Science, University of the Ryukyus, 1 Senbaru, Nishihara, Okinawa, 903-0213, Japan

^4^Tropical Biosphere Research Center, University of the Ryukyus, 1 Senbaru, Nishihara, Okinawa, 903-0213, Japan

^5^School of Environmental and Life Sciences, The University of Newcastle, Callaghan, NSW, 2308, Australia

^6^School of Biology, Faculty of Biological Sciences, University of Leeds, Leeds, LS2 9JT, United Kingdom

^7^School of Biological Sciences, Victoria University of Wellington, PO Box 600, Wellington, 6140, New Zealand

^8^Department of Biological Sciences, Macquarie University, North Ryde, NSW, 2113, Australia

^9^Environmental Protection Authority, 215 Lambton Quay, Wellington, 6011, New Zealand

**Table S2.** List of all combined families assigned in MEGAN v 5.11.3, including order, class, and phylum based on 18S rRNA sequences from sediment and seawater collected at 14 sites off the coast of Okinawa, Japan. All assignments are based on our DNA sequence quality filter criteria outlined in the Methods.

| Family | Order | Class | Phylum |
| --- | --- | --- | --- |
| Acanthaceae | Lamiales | Magnoliopsida | Tracheophyta |
| Acanthochiasmidae | Holacanthida | Acantharia | Radiozoa |
| Acanthochitonidae | Chitonida | Polyplacophora | Mollusca |
| Acanthocystidae | Centrohelida | Centrohelea | Heliozoa |
| Acartiidae | Calanoida | Hexanauplia | Arthropoda |
| Achnanthaceae | Achnanthales | Bacillariophyceae | Ochrophyta |
| Acinetidae | Endogenida | Phyllopharyngea | Ciliophora |
| Acroporidae | Scleractinia | Anthozoa | Cnidaria |
| Actiniidae | Actiniaria | Anthozoa | Cnidaria |
| Aeginidae | Narcomedusae | Hydrozoa | Cnidaria |
| Aetideidae | Calanoida | Hexanauplia | Arthropoda |
| Agalmatidae | Siphonophorae | Hydrozoa | Cnidaria |
| Aglajidae | Cephalaspidea | Gastropoda | Mollusca |
| Aglaopheniidae | Leptothecata | Hydrozoa | Cnidaria |
| Amaryllidaceae | Asparagales | Magnoliopsida | Tracheophyta |
| Amoebophryaceae | Syndiniales | Dinophyceae | Myzozoa |
| Amphidomataceae | Dinophyceae incertae sedis | Dinophyceae | Myzozoa |
| Amphileptidae | Pleurostomatida | Litostomatea | Ciliophora |
| Amphinomidae | Amphinomida | Polychaeta | Annelida |
| Amphiporidae | Monostilifera | Enopla | Nemertea |
| Amphisphaeriaceae | Xylariales | Sordariomycetes | Ascomycota |
| Anadyomenaceae | Cladophorales | Ulvophyceae | Chlorophyta |
| Anchimolgidae | Cyclopoida | Hexanauplia | Arthropoda |
| Ancorinidae | Tetractinellida | Demospongiae | Porifera |
| Anthessiidae | Cyclopoida | Hexanauplia | Arthropoda |
| Aphroditidae | Phyllodocida | Polychaeta | Annelida |
| Aplysiidae | Aplysiida | Gastropoda | Mollusca |
| Apusomonadidae | Apusomonadida | Thecomonadea | Apusozoa |
| Arcidae | Arcida | Bivalvia | Mollusca |
| Arenicolidae |  | Polychaeta | Annelida |
| Armadillidiidae | Isopoda | Malacostraca | Arthropoda |
| Ascidiidae | Phlebobranchia | Ascidiacea | Chordata |
| Aspergillaceae | Eurotiales | Eurotiomycetes | Ascomycota |
| Aspidiscidae | Euplotida | Spirotrichea | Ciliophora |
| Asterocheridae | Siphonostomatoida | Hexanauplia | Arthropoda |
| Aureobasidiaceae | Dothideales | Dothideomycetes | Ascomycota |
| Axiidae | Decapoda | Malacostraca | Arthropoda |
| Bacillariaceae | Bacillariales | Bacillariophyceae | Ochrophyta |
| Bangiaceae | Bangiales | Bangiophyceae | Rhodophyta |
| Bathycoccaceae | Mamiellales | Mamiellophyceae | Chlorophyta |
| Berkeleyaceae | Naviculales | Bacillariophyceae | Ochrophyta |
| Bicosoecidae | Bicoecida | Bicoecea | Bigyra |
| Biddulphiaceae | Biddulphiales | Bacillariophyceae | Ochrophyta |
| Biecheleriaceae | Suessiales | Dinophyceae | Myzozoa |
| Blastocladiaceae | Blastocladiales | Blastocladiomycetes | Blastocladiomycota |
| Blattidae | Blattodea | Insecta | Arthropoda |
| Blepharismidae | Heterotrichida | Heterotrichea | Ciliophora |
| Borghiellaceae | Suessiales | Dinophyceae | Myzozoa |
| Bryopsidaceae | Bryopsidales | Ulvophyceae | Chlorophyta |
| Buccinidae | Neogastropoda | Gastropoda | Mollusca |
| Cafeteriaceae | Bicoecida | Bicoecea | Bigyra |
| Calanidae | Calanoida | Hexanauplia | Arthropoda |
| Calcidiscaceae | Coccolithales | Prymnesiophyceae | Haptophyta |
| Callochitonidae | Chitonida | Polyplacophora | Mollusca |
| Calloporidae | Cheilostomatida | Gymnolaemata | Bryozoa |
| Callyspongiidae | Haplosclerida | Demospongiae | Porifera |
| Campanulariidae | Leptothecata | Hydrozoa | Cnidaria |
| Canthocamptidae | Harpacticoida | Hexanauplia | Arthropoda |
| Capitellidae |  | Polychaeta | Annelida |
| Cardiidae | Cardiida | Bivalvia | Mollusca |
| Catenulaceae | Thalassiophysales | Bacillariophyceae | Ochrophyta |
| Catiniidae | Cyclopoida | Hexanauplia | Arthropoda |
| Caulacanthaceae | Gigartinales | Florideophyceae | Rhodophyta |
| Caulerpaceae | Bryopsidales | Ulvophyceae | Chlorophyta |
| Centropagidae | Calanoida | Hexanauplia | Arthropoda |
| Cephalotrichidae |  | Palaeonemertea | Nemertea |
| Ceramiaceae | Ceramiales | Florideophyceae | Rhodophyta |
| Ceratiaceae | Gonyaulacales | Dinophyceae | Myzozoa |
| Ceratomyxidae | Bivalvulida | Myxozoa | Cnidaria |
| Cercomonadidae | Cercomonadida | Sarcomonadea | Cercozoa |
| Chaetocerotaceae | Chaetocerotanae incertae sedis | Bacillariophyceae | Ochrophyta |
| Chaetonotidae | Chaetonotida |  | Gastrotricha |
| Chaetopteridae |  | Polychaeta | Annelida |
| Chalinidae | Haplosclerida | Demospongiae | Porifera |
| Chamidae |  | Bivalvia | Mollusca |
| Chattonellaceae | Chattonellales | Raphidophyceae | Ochrophyta |
| Chitonidae | Chitonida | Polyplacophora | Mollusca |
| Chlamydophryidae | Tectofilosida | Thecofilosea | Cercozoa |
| Chlorellaceae | Chlorellales | Trebouxiophyceae | Chlorophyta |
| Chlorodendraceae | Chlorodendrales | Chlorodendrophyceae | Chlorophyta |
| Chondrillidae | Chondrillida | Demospongiae | Porifera |
| Chromadoridae | Chromadorida | Chromadorea | Nematoda |
| Chromodorididae | Nudibranchia | Gastropoda | Mollusca |
| Chroomonadaceae | Pyrenomonadales | Cryptophyceae | Cryptophyta |
| Chrysochromulinaceae | Prymnesiales | Prymnesiophyceae | Haptophyta |
| Chrysolepidomonadaceae | Chromulinales | Chrysophyceae | Ochrophyta |
| Chrysomeridaceae | Chrysomeridales | Chrysomerophyceae | Ochrophyta |
| Chrysopetalidae | Phyllodocida | Polychaeta | Annelida |
| Chytridiaceae | Chytridiales | Chytridiomycetes | Chytridiomycota |
| Chytriodiniaceae | Coccidiniales | Dinophyceae | Myzozoa |
| Chytriomycetaceae | Chytridiales | Chytridiomycetes | Chytridiomycota |
| Cinetochilidae | Philasterida | Oligohymenophorea | Ciliophora |
| Cirratulidae | Terebellida | Polychaeta | Annelida |
| Cladophoraceae | Cladophorales | Ulvophyceae | Chlorophyta |
| Cladosporiaceae | Capnodiales | Dothideomycetes | Ascomycota |
| Clausidiidae | Cyclopoida | Hexanauplia | Arthropoda |
| Clausocalanidae | Calanoida | Hexanauplia | Arthropoda |
| Clavelinidae | Aplousobranchia | Ascidiacea | Chordata |
| Hydractiniidae | Anthoathecata | Hydrozoa | Cnidaria |
| Clionaidae | Clionaida | Demospongiae | Porifera |
| Clupeidae | Clupeiformes | Actinopterygii | Chordata |
| Cocconeidaceae | Achnanthales | Bacillariophyceae | Ochrophyta |
| Codosigidae | Craspedida | Choanoflagellatea | Choanozoa |
| Cohnilembidae | Philasterida | Oligohymenophorea | Ciliophora |
| Colepidae | Prorodontida | Prostomatea | Ciliophora |
| Colpodellidae | Colpodellida | Apicomonadea | Myzozoa |
| Condylostomatidae | Heterotrichida | Heterotrichea | Ciliophora |
| Conidae | Neogastropoda | Gastropoda | Mollusca |
| Corallinaceae | Corallinales | Florideophyceae | Rhodophyta |
| Corethraceae | Corethrales | Bacillariophyceae | Ochrophyta |
| Corynidae | Anthoathecata | Hydrozoa | Cnidaria |
| Creseidae | Pteropoda | Gastropoda | Mollusca |
| Cryptoplacidae | Chitonida | Polyplacophora | Mollusca |
| Cucumariidae | Dendrochirotida | Holothuroidea | Echinodermata |
| Cushmanideidae | Podocopida | Ostracoda | Arthropoda |
| Cyanophoraceae | Cyanophorales | Glaucophyceae | Glaucophyta |
| Cyatholaimidae | Chromadorida | Chromadorea | Nematoda |
| Cyclidiidae | Pleuronematida | Oligohymenophorea | Ciliophora |
| Cyclopettidae | Cyclopoida | Hexanauplia | Arthropoda |
| Cyclophoraceae | Cyclophorales | Bacillariophyceae | Ochrophyta |
| Cyclopidae | Cyclopoida | Hexanauplia | Arthropoda |
| Cyclopinidae | Cyclopoida | Hexanauplia | Arthropoda |
| Cymatosiraceae | Cymatosirales | Bacillariophyceae | Ochrophyta |
| Cyprididae | Podocopida | Ostracoda | Arthropoda |
| Cypridinidae | Myodocopida | Ostracoda | Arthropoda |
| Cyrenidae | Venerida | Bivalvia | Mollusca |
| Cyrtostrombidiidae | Oligotrichida | Oligotrichea | Ciliophora |
| Cystiplanidae | Rhabdocoela | Rhabditophora | Platyhelminthes |
| Cystocloniaceae | Gigartinales | Florideophyceae | Rhodophyta |
| Cystofilobasidiaceae | Cystofilobasidiales | Tremellomycetes | Basidiomycota |
| Cytheruridae | Podocopida | Ostracoda | Arthropoda |
| Dactylopusiidae | Harpacticoida | Hexanauplia | Arthropoda |
| Darwinellidae | Dendroceratida | Demospongiae | Porifera |
| Dendrophylliidae | Scleractinia | Anthozoa | Cnidaria |
| Densiporidae | Cyclostomatida | Stenolaemata | Bryozoa |
| Desmacellidae | Desmacellida | Demospongiae | Porifera |
| Desmodoridae | Desmodorida | Chromadorea | Nematoda |
| Desmoscolecidae | Desmoscolecida | Chromadorea | Nematoda |
| Diadesmidaceae | Naviculales | Bacillariophyceae | Ochrophyta |
| Dictyodendrillidae | Dendroceratida | Demospongiae | Porifera |
| Dictyonellidae | Bubarida | Demospongiae | Porifera |
| Dictyotaceae | Dictyotales | Phaeophyceae | Ochrophyta |
| Didemnidae | Aplousobranchia | Ascidiacea | Chordata |
| Didiniidae | Haptorida | Litostomatea | Ciliophora |
| Didymellaceae | Pleosporales | Dothideomycetes | Ascomycota |
| Didymosphaeriaceae | Pleosporales | Dothideomycetes | Ascomycota |
| Diploneidaceae | Naviculales | Bacillariophyceae | Ochrophyta |
| Discocephalidae | Euplotida | Spirotrichea | Ciliophora |
| Dolichomacrostomidae | Dolichomicrostomida | Rhabditophora | Platyhelminthes |
| Dolichomastigaceae | Dolichomastigales | Mamiellophyceae | Chlorophyta |
| Donacidae | Cardiida | Bivalvia | Mollusca |
| Dorataspididae | Arthracanthida | Acantharia | Radiozoa |
| Dorvilleidae | Eunicida | Polychaeta | Annelida |
| Draconematidae | Desmodorida | Chromadorea | Nematoda |
| Dromiidae | Decapoda | Malacostraca | Arthropoda |
| Duboscquellidae | Syndiniales | Dinophyceae | Myzozoa |
| Echiuridae | Echiuroidea | Polychaeta | Annelida |
| Ectinosomatidae | Harpacticoida | Hexanauplia | Arthropoda |
| Eimeriidae | Eucoccidiorida | Conoidasida | Myzozoa |
| Elachisinidae | Littorinimorpha | Gastropoda | Mollusca |
| Emplectonematidae | Monostilifera | Enopla | Nemertea |
| Enoplidae | Enoplida | Enoplea | Nematoda |
| Entomolepididae | Siphonostomatoida | Hexanauplia | Arthropoda |
| Entomoneidaceae | Surirellales | Bacillariophyceae | Ochrophyta |
| Ephelotidae | Exogenida | Phyllopharyngea | Ciliophora |
| Epiclintidae |  | Spirotrichea | Ciliophora |
| Erythropeltidaceae | Erythropeltidales | Compsopogonophyceae | Rhodophyta |
| Erythrotrichiaceae | Erythropeltidales | Compsopogonophyceae | Rhodophyta |
| Eucytheridae | Podocopida | Ostracoda | Arthropoda |
| Eudubosquellidae | Syndiniales | Dinophyceae | Myzozoa |
| Eulimidae | Littorinimorpha | Gastropoda | Mollusca |
| Eunicidae | Eunicida | Polychaeta | Annelida |
| Eunotiaceae | Eunotiales | Bacillariophyceae | Ochrophyta |
| Euplotidae | Euplotida | Spirotrichea | Ciliophora |
| Exobasidiaceae | Exobasidiales | Exobasidiomycetes | Basidiomycota |
| Fabaceae | Fabales | Magnoliopsida | Tracheophyta |
| Fabriciidae | Sabellida | Polychaeta | Annelida |
| Fissurellidae | Lepetellida | Gastropoda | Mollusca |
| Flabellulidae | Leptomyxida | Tubulinea | Amoebozoa |
| Folliculinidae | Heterotrichida | Heterotrichea | Ciliophora |
| Fragilariaceae | Fragilariales | Bacillariophyceae | Ochrophyta |
| Frontoniidae | Peniculida | Oligohymenophorea | Ciliophora |
| Gastrochaenidae |  | Bivalvia | Mollusca |
| Gastropteridae | Cephalaspidea | Gastropoda | Mollusca |
| Geleiidae | Protoheterotrichida | Karyorelictea | Ciliophora |
| Gelidiaceae | Gelidiales | Florideophyceae | Rhodophyta |
| Geminigeraceae | Pyrenomonadales | Cryptophyceae | Cryptophyta |
| Geodiidae | Tetractinellida | Demospongiae | Porifera |
| Geryoniidae | Trachymedusae | Hydrozoa | Cnidaria |
| Gigartinaceae | Gigartinales | Florideophyceae | Rhodophyta |
| Glenodiniaceae | Peridiniales | Dinophyceae | Myzozoa |
| Glomerellaceae | Hypocreomycetidae incertae sedis | Sordariomycetes | Ascomycota |
| Glyceridae | Phyllodocida | Polychaeta | Annelida |
| Goniodomataceae | Gonyaulacales | Dinophyceae | Myzozoa |
| Goniomonadaceae | Goniomonadales | Goniomonadophyceae | Cryptophyta |
| Gonyaulacaceae | Gonyaulacales | Dinophyceae | Myzozoa |
| Grantiidae | Leucosolenida | Calcarea | Porifera |
| Graphiolaceae | Exobasidiales | Exobasidiomycetes | Basidiomycota |
| Gregarinidae | Eugregarinorida | Conoidasida | Myzozoa |
| Gryllidae | Orthoptera | Insecta | Arthropoda |
| Gymnodiniaceae | Gymnodiniales | Dinophyceae | Myzozoa |
| Halacaridae | Trombidiformes | Arachnida | Arthropoda |
| Haleciidae | Leptothecata | Hydrozoa | Cnidaria |
| Halichondriidae | Suberitida | Demospongiae | Porifera |
| Haliphthoraceae | Saprolegniales | Peronosporea | Oomycota |
| Halisarcidae | Chondrillida | Demospongiae | Porifera |
| Halteriidae | Halteriida | Oligotrichea | Ciliophora |
| Halymeniaceae | Halymeniales | Florideophyceae | Rhodophyta |
| Haminoeidae | Cephalaspidea | Gastropoda | Mollusca |
| Hapalidiaceae | Corallinales | Florideophyceae | Rhodophyta |
| Harpacticidae | Harpacticoida | Hexanauplia | Arthropoda |
| Hausmanniellidae | Colpodida | Colpodea | Ciliophora |
| Helminthosphaeriaceae | Trichosphaeriales | Sordariomycetes | Ascomycota |
| Hemiaulaceae | Hemiaulales | Bacillariophyceae | Ochrophyta |
| Hemidiscaceae | Coscinodiscales | Bacillariophyceae | Ochrophyta |
| Hemiuridae | Plagiorchiida | Trematoda | Platyhelminthes |
| Herpotrichiellaceae | Chaetothyriales | Eurotiomycetes | Ascomycota |
| Hesionidae | Phyllodocida | Polychaeta | Annelida |
| Heterocapsaceae | Peridiniales | Dinophyceae | Myzozoa |
| Heteromitidae | Cercomonadida | Sarcomonadea | Cercozoa |
| Heteroporidae | Cyclostomatida | Stenolaemata | Bryozoa |
| Holostichidae |  | Spirotrichea | Ciliophora |
| Holothuriidae | Holothuriida | Holothuroidea | Echinodermata |
| Horneridae | Cyclostomatida | Stenolaemata | Bryozoa |
| Hymedesmiidae | Poecilosclerida | Demospongiae | Porifera |
| Hypocreaceae | Hypocreales | Sordariomycetes | Ascomycota |
| Iotrochotidae | Poecilosclerida | Demospongiae | Porifera |
| Ironidae | Enoplida | Enoplea | Nematoda |
| Ischnochitonidae | Chitonida | Polyplacophora | Mollusca |
| Isodiametridae | Acoela |  | Xenacoelomorpha |
| Jakobidae | Jakobida | Jakobea | Loukozoa |
| Kareniaceae | Gymnodiniales | Dinophyceae | Myzozoa |
| Karkinorhynchidae | Rhabdocoela | Rhabditophora | Platyhelminthes |
| Katablepharidaceae |  | Cryptophyta incertae sedis | Cryptophyta |
| Koralionastetaceae | Sordariomycetes incertae sedis | Sordariomycetes | Ascomycota |
| Labridae | Perciformes | Actinopterygii | Chordata |
| Labyrinthula | Labyrinthulida | Labyrinthulae | Bigyra |
| Lacrymariidae | Haptorida | Litostomatea | Ciliophora |
| Lasaeidae |  | Bivalvia | Mollusca |
| Lecudinidae | Eugregarinorida | Conoidasida | Myzozoa |
| Lepidodasyidae | Macrodasyida |  | Gastrotricha |
| Leptochitonidae | Lepidopleurida | Polyplacophora | Mollusca |
| Leptocylindraceae | Leptocylindrales | Bacillariophyceae | Ochrophyta |
| Leptocytheridae | Podocopida | Ostracoda | Arthropoda |
| Leptolaimidae | Plectida | Chromadorea | Nematoda |
| Liagoraceae | Nemaliales | Florideophyceae | Rhodophyta |
| Lichenoporidae | Cyclostomatida | Stenolaemata | Bryozoa |
| Lichinaceae | Lichinales | Lichinomycetes | Ascomycota |
| Lichomolgidae | Cyclopoida | Hexanauplia | Arthropoda |
| Licmophoraceae | Licmophorales | Bacillariophyceae | Ochrophyta |
| Licnophoridae | Licnophorida | Spirotrichea | Ciliophora |
| Limidae | Limida | Bivalvia | Mollusca |
| Lineidae |  | Anopla | Nemertea |
| Linyphiidae | Araneae | Arachnida | Arthropoda |
| Lithoglyptidae | Lithoglyptida | Hexanauplia | Arthropoda |
| Litonotidae | Pleurostomatida | Litostomatea | Ciliophora |
| Littorinidae | Littorinimorpha | Gastropoda | Mollusca |
| Lobulomycetaceae | Lobulomycetales | Lobulomycetes | Chytridiomycota |
| Lomentariaceae | Rhodymeniales | Florideophyceae | Rhodophyta |
| Lottiidae |  | Gastropoda | Mollusca |
| Loxoconchidae | Podocopida | Ostracoda | Arthropoda |
| Lucinidae | Lucinida | Bivalvia | Mollusca |
| Lulworthiaceae | Lulworthiales | Sordariomycetes | Ascomycota |
| Lycosidae | Araneae | Arachnida | Arthropoda |
| Lynnellidae | Choreotrichida | Oligotrichea | Ciliophora |
| Macrostomidae |  | Rhabditophora | Platyhelminthes |
| Magnaporthaceae | Magnaporthales | Sordariomycetes | Ascomycota |
| Maldanidae |  | Polychaeta | Annelida |
| Malleidae | Ostreida | Bivalvia | Mollusca |
| Mamiellaceae | Mamiellales | Mamiellophyceae | Chlorophyta |
| Mantamonadidae |  |  | Apusozoa |
| Marsupiomonadaceae | Marsupiomonadales | Pedinophyceae | Chlorophyta |
| Mecynostomidae | Acoela |  | Xenacoelomorpha |
| Membracidae | Hemiptera | Insecta | Arthropoda |
| Membraniporidae | Cheilostomatida | Gymnolaemata | Bryozoa |
| Merulinidae | Scleractinia | Anthozoa | Cnidaria |
| Mesodiniidae | Cyclotrichiida | Litostomatea | Ciliophora |
| Microcionidae | Poecilosclerida | Demospongiae | Porifera |
| Microlaimidae | Desmodorida | Chromadorea | Nematoda |
| Microstomidae | Dolichomicrostomida | Rhabditophora | Platyhelminthes |
| Microthoracidae | Microthoracida | Nassophorea | Ciliophora |
| Milleporidae | Anthoathecata | Hydrozoa | Cnidaria |
| Miraciidae | Harpacticoida | Hexanauplia | Arthropoda |
| Molgulidae | Stolidobranchia | Ascidiacea | Chordata |
| Monocelididae | Proseriata | Rhabditophora | Platyhelminthes |
| Monodopsidaceae | Eustigmatales | Eustigmatophyceae | Ochrophyta |
| Monostromataceae | Ulvales | Ulvophyceae | Chlorophyta |
| Mugilidae | Perciformes | Actinopterygii | Chordata |
| Mycalidae | Poecilosclerida | Demospongiae | Porifera |
| Mytilidae | Mytilida | Bivalvia | Mollusca |
| Naididae | Haplotaxida | Clitellata | Annelida |
| Naviculaceae | Naviculales | Bacillariophyceae | Ochrophyta |
| Nectriaceae | Hypocreales | Sordariomycetes | Ascomycota |
| Nemastomataceae | Nemastomatales | Florideophyceae | Rhodophyta |
| Nereididae | Phyllodocida | Polychaeta | Annelida |
| Nerillidae |  | Polychaeta | Annelida |
| Neritidae | Cycloneritida | Gastropoda | Mollusca |
| Niphatidae | Haplosclerida | Demospongiae | Porifera |
| Oikopleuridae | Copelata | Appendicularia | Chordata |
| Oithonidae | Cyclopoida | Hexanauplia | Arthropoda |
| Oncholaimidae | Enoplida | Enoplea | Nematoda |
| Opheliidae |  | Polychaeta | Annelida |
| Ophiodermatidae | Ophiacanthida | Ophiuroidea | Echinodermata |
| Orbiniidae |  | Polychaeta | Annelida |
| Orchitophryidae | Philasterida | Oligohymenophorea | Ciliophora |
| Orthodonellidae | Synhymeniida | Nassophorea | Ciliophora |
| Ostreidae | Ostreida | Bivalvia | Mollusca |
| Ostreopsidaceae | Gonyaulacales | Dinophyceae | Myzozoa |
| Otoplanidae | Proseriata | Rhabditophora | Platyhelminthes |
| Oweniidae | Sabellida | Polychaeta | Annelida |
| Oxytrichidae |  | Spirotrichea | Ciliophora |
| Paracalanidae | Calanoida | Hexanauplia | Arthropoda |
| Paradrepanophoridae | Polystilifera | Enopla | Nemertea |
| Parameciidae | Peniculida | Oligohymenophorea | Ciliophora |
| Paramoebidae | Dactylopodida | Discosea | Amoebozoa |
| Paraonidae |  | Polychaeta | Annelida |
| Paraphysomonadaceae | Ochromonadales | Chrysophyceae | Ochrophyta |
| Parastenheliidae | Harpacticoida | Hexanauplia | Arthropoda |
| Parazoanthidae | Zoantharia | Anthozoa | Cnidaria |
| Parhedylidae |  | Gastropoda | Mollusca |
| Pavlovaceae | Pavlovales | Pavlovophyceae | Haptophyta |
| Pectinidae | Pectinida | Bivalvia | Mollusca |
| Pedinomonadaceae | Pedinomonadales | Pedinophyceae | Chlorophyta |
| Peridiniaceae | Peridiniales | Dinophyceae | Myzozoa |
| Peritromidae | Heterotrichida | Heterotrichea | Ciliophora |
| Perkinsidae | Perkinsida | Perkinsea | Myzozoa |
| Perophoridae | Phlebobranchia | Ascidiacea | Chordata |
| Petrosiidae | Haplosclerida | Demospongiae | Porifera |
| Peyssonneliaceae | Peyssonneliales | Florideophyceae | Rhodophyta |
| Pfiesteriaceae | Peridiniales | Dinophyceae | Myzozoa |
| Phaeocystaceae | Phaeocystales | Prymnesiophyceae | Haptophyta |
| Phaeophilaceae | Ulvales | Ulvophyceae | Chlorophyta |
| Phascolosomatidae | Phascolosomatida | Phascolosomatidea | Sipuncula |
| Philasteridae | Philasterida | Oligohymenophorea | Ciliophora |
| Philinoglossidae | Cephalaspidea | Gastropoda | Mollusca |
| Phloeodictyidae | Haplosclerida | Demospongiae | Porifera |
| Pholoidae | Phyllodocida | Polychaeta | Annelida |
| Phyllodocidae | Phyllodocida | Polychaeta | Annelida |
| Physidae |  | Gastropoda | Mollusca |
| Picomonadidae | Picomonadida | Picomonadea | Picozoa |
| Pinaceae | Pinales | Pinopsida | Tracheophyta |
| Pinguiochrysidaceae | Pinguiochrysidales | Dictyochophyceae | Ochrophyta |
| Pinnidae | Ostreida | Bivalvia | Mollusca |
| Pinnulariaceae | Naviculales | Bacillariophyceae | Ochrophyta |
| Placorhynchidae | Rhabdocoela | Rhabditophora | Platyhelminthes |
| Placospongiidae | Clionaida | Demospongiae | Porifera |
| Plagiacanthidae | Nassellaria | Polycystina | Radiozoa |
| Plagiogrammaceae | Triceratiales | Bacillariophyceae | Ochrophyta |
| Plakinidae | Homosclerophorida | Homoscleromorpha | Porifera |
| Planomonadidae | Planomonadida | Hilomonadea | Apusozoa |
| Plasmodiophoridae | Plasmodiophorida | Phytomyxea | Cercozoa |
| Plectosphaerellaceae | Hypocreomycetidae incertae sedis | Sordariomycetes | Ascomycota |
| Pleosporaceae | Pleosporales | Dothideomycetes | Ascomycota |
| Pleurobranchidae | Pleurobranchomorpha | Gastropoda | Mollusca |
| Pleuronematidae | Pleuronematida | Oligohymenophorea | Ciliophora |
| Pleurosigmataceae | Naviculales | Bacillariophyceae | Ochrophyta |
| Plicatulidae | Pectinida | Bivalvia | Mollusca |
| Plumulariidae | Leptothecata | Hydrozoa | Cnidaria |
| Pocilloporidae | Scleractinia | Anthozoa | Cnidaria |
| Poecilochaetidae | Spionida | Polychaeta | Annelida |
| Polyclinidae | Aplousobranchia | Ascidiacea | Chordata |
| Polycystididae | Rhabdocoela | Rhabditophora | Platyhelminthes |
| Polynoidae | Phyllodocida | Polychaeta | Annelida |
| Polyphysaceae | Dasycladales | Ulvophyceae | Chlorophyta |
| Pomacanthidae | Perciformes | Actinopterygii | Chordata |
| Pomacentridae | Perciformes | Actinopterygii | Chordata |
| Pontellidae | Calanoida | Hexanauplia | Arthropoda |
| Porphyridiaceae | Porphyridiales | Porphyridiophyceae | Rhodophyta |
| Porpitidae | Anthoathecata | Hydrozoa | Cnidaria |
| Promesostomidae | Rhabdocoela | Rhabditophora | Platyhelminthes |
| Prorocentraceae | Prorocentrales | Dinophyceae | Myzozoa |
| Protodrilidae |  | Polychaeta | Annelida |
| Protoperidiniaceae | Peridiniales | Dinophyceae | Myzozoa |
| Prymnesiaceae | Prymnesiales | Prymnesiophyceae | Haptophyta |
| Pseudanthessiidae | Cyclopoida | Hexanauplia | Arthropoda |
| Pseudocohnilembidae | Philasterida | Oligohymenophorea | Ciliophora |
| Pseudodiaptomidae | Calanoida | Hexanauplia | Arthropoda |
| Pseudokeronopsidae |  | Spirotrichea | Ciliophora |
| Pseudourostylidae |  | Spirotrichea | Ciliophora |
| Pteriidae | Ostreida | Bivalvia | Mollusca |
| Ptychoderidae | [unassigned] Enteropneusta | Enteropneusta | Hemichordata |
| Pycnococcaceae | Pseudoscourfieldiales | Pyramimonadophyceae | Chlorophyta |
| Pyramidellidae |  | Gastropoda | Mollusca |
| Pyrenomonadaceae | Pyrenomonadales | Cryptophyceae | Cryptophyta |
| Pyrgomatidae | Sessilia | Hexanauplia | Arthropoda |
| Pyrophaceae | Peridinales | Dinophyceae | Myzozoa |
| Pythiaceae | Pythiales | Peronosporea | Oomycota |
| Pyuridae | Stolidobranchia | Ascidiacea | Chordata |
| Raspailiidae | Axinellida | Demospongiae | Porifera |
| Reduviidae | Hemiptera | Insecta | Arthropoda |
| Rhabdonemataceae | Rhabdonematales | Bacillariophyceae | Ochrophyta |
| Rhaphoneidaceae | Rhaponeidales | Bacillariophyceae | Ochrophyta |
| Rhizophyllidaceae | Gigartinales | Florideophyceae | Rhodophyta |
| Rhizosoleniaceae | Rhizosoleniales | Bacillariophyceae | Ochrophyta |
| Rhodellaceae | Rhodellales | Rhodellophyceae | Rhodophyta |
| Rhodochaetaceae | Rhodochaetales | Compsopogonophyceae | Rhodophyta |
| Rhodomelaceae | Ceramiales | Florideophyceae | Rhodophyta |
| Rhodymeniaceae | Rhodymeniales | Florideophyceae | Rhodophyta |
| Rhopalodiaceae | Rhopalodiales | Bacillariophyceae | Ochrophyta |
| Rhopalonematidae | Trachymedusae | Hydrozoa | Cnidaria |
| Romancheinidae | Cheilostomatida | Gymnolaemata | Bryozoa |
| Sabellariidae | Canalipalpata | Polychaeta | Annelida |
| Sabellidae | Sabellida | Polychaeta | Annelida |
| Salmonidae | Salmoniformes | Actinopterygii | Chordata |
| Salpidae | Salpida | Thaliacea | Chordata |
| Sapphirinidae | Cyclopoida | Hexanauplia | Arthropoda |
| Sargassaceae | Fucales | Phaeophyceae | Ochrophyta |
| Scalibregmatidae |  | Polychaeta | Annelida |
| Scenedesmaceae | Sphaeropleales | Chlorophyceae | Chlorophyta |
| Schizorhynchidae | Rhabdocoela | Rhabditophora | Platyhelminthes |
| Schizymeniaceae | Nemastomatales | Florideophyceae | Rhodophyta |
| Scleritodermidae | Tetractinellida | Demospongiae | Porifera |
| Sclerodactylidae | Dendrochirotida | Holothuroidea | Echinodermata |
| Sclerotiniaceae | Helotiales | Leotiomycetes | Ascomycota |
| Scoliotropidaceae | Naviculales | Bacillariophyceae | Ochrophyta |
| Scotinosphaeraceae | Scotinosphaerales | Ulvophyceae | Chlorophyta |
| Selenidiidae | Eugregarinorida | Conoidasida | Myzozoa |
| Sellaphoraceae | Naviculales | Bacillariophyceae | Ochrophyta |
| Serpulidae | Sabellida | Polychaeta | Annelida |
| Sertulariidae | Leptothecata | Hydrozoa | Cnidaria |
| Sigalionidae | Phyllodocida | Polychaeta | Annelida |
| Siganidae | Perciformes | Actinopterygii | Chordata |
| Sipunculidae | Golfingiida | Sipunculidea | Sipuncula |
| Sirobasidiaceae | Tremellales | Tremellomycetes | Basidiomycota |
| Skeletonemaceae | Thalassiosirales | Bacillariophyceae | Ochrophyta |
| Haminoeidae | Cephalaspidea | Gastropoda | Mollusca |
| Solanaceae | Solanales | Magnoliopsida | Tracheophyta |
| Solenopharyngidae | Rhabdocoela | Rhabditophora | Platyhelminthes |
| Spengelidae | [unassigned] Enteropneusta | Enteropneusta | Hemichordata |
| Sphinctrinaceae | Mycocaliciales | Eurotiomycetes | Ascomycota |
| Spionidae | Spionida | Polychaeta | Annelida |
| Spirastrellidae | Clionaida | Demospongiae | Porifera |
| Spirostomidae | Heterotrichida | Heterotrichea | Ciliophora |
| Spondylidae | Pectinida | Bivalvia | Mollusca |
| Spongodiscidae | Spumellaria | Polycystina | Radiozoa |
| Sporolithaceae | Corallinales | Florideophyceae | Rhodophyta |
| Stemonitidae | Stemonitida | Myxogastrea | Amoebozoa |
| Stentoridae | Heterotrichida | Heterotrichea | Ciliophora |
| Stephanodiscaceae | Thalassiosirales | Bacillariophyceae | Ochrophyta |
| Striatellaceae | Striatellales | Bacillariophyceae | Ochrophyta |
| Strobilidiidae | Choreotrichida | Oligotrichea | Ciliophora |
| Strombidae | Littorinimorpha | Gastropoda | Mollusca |
| Strombidiidae | Oligotrichida | Oligotrichea | Ciliophora |
| Strombidinopsidae | Choreotrichida | Oligotrichea | Ciliophora |
| Styelidae | Stolidobranchia | Ascidiacea | Chordata |
| Stylonemataceae | Stylonematales | Stylonematophyceae | Rhodophyta |
| Suberitidae | Suberitida | Demospongiae | Porifera |
| Sycettidae | Leucosolenida | Calcarea | Porifera |
| Syllidae | Phyllodocida | Polychaeta | Annelida |
| Symbiodiniaceae | Suessiales | Dinophyceae | Myzozoa |
| Synaptidae | Apodida | Holothuroidea | Echinodermata |
| Syndiniaceae | Coccidiniales | Dinophyceae | Myzozoa |
| Syracosphaeraceae | Syracosphaerales | Prymnesiophyceae | Haptophyta |
| Tellinidae | Cardiida | Bivalvia | Mollusca |
| Teratocephalidae | Rhabditida | Chromadorea | Nematoda |
| Terebellidae | Terebellida | Polychaeta | Annelida |
| Teredinidae | Myida | Bivalvia | Mollusca |
| Tethyidae | Tethyida | Demospongiae | Porifera |
| Tetillidae | Tetractinellida | Demospongiae | Porifera |
| Thaerocytheridae | Podocopida | Ostracoda | Arthropoda |
| Thalassionemataceae | Thalassionematales | Bacillariophyceae | Ochrophyta |
| Thalassiosiraceae | Thalassiosirales | Bacillariophyceae | Ochrophyta |
| Thalestridae | Harpacticoida | Hexanauplia | Arthropoda |
| Thaumastodermatidae | Macrodasyida |  | Gastrotricha |
| Thaumatomonadidae | Thaumatomonadida | Imbricatea | Cercozoa |
| Theonellidae | Tetractinellida | Demospongiae | Porifera |
| Thoracosphaeraceae | Thoracosphaerales | Dinophyceae | Myzozoa |
| Thoracostomopsidae | Enoplida | Enoplea | Nematoda |
| Thorectidae | Dictyoceratida | Demospongiae | Porifera |
| Thraustochytriaceae | Thraustochytrida | Labyrinthulea | Bigyra |
| Thripidae | Thysanoptera | Insecta | Arthropoda |
| Thyasiridae | Lucinida | Bivalvia | Mollusca |
| Tilletiaceae | Tilletiales | Ustilaginomycetes | Basidiomycota |
| Timeidae | Tethyida | Demospongiae | Porifera |
| Tintinnidae | Choreotrichida | Oligotrichea | Ciliophora |
| Tontoniidae | Oligotrichida | Oligotrichea | Ciliophora |
| Trachelocercidae | Protostomatida | Karyorelictea | Ciliophora |
| Trachelostylidae | Sporadotrichida | Spirotrichea | Ciliophora |
| Trigonostomidae | Rhabdocoela | Rhabditophora | Platyhelminthes |
| Tubulanidae |  | Palaeonemertea | Nemertea |
| Turbinidae | Trochida | Gastropoda | Mollusca |
| Ulotrichaceae | Ulotrichales | Ulvophyceae | Chlorophyta |
| Ulvaceae | Ulvales | Ulvophyceae | Chlorophyta |
| Ulvellaceae | Ulvales | Ulvophyceae | Chlorophyta |
| Uronematidae | Philasterida | Oligohymenophorea | Ciliophora |
| Uronychiidae | Euplotida | Spirotrichea | Ciliophora |
| Ustilaginaceae | Ustilaginales | Ustilaginomycetes | Basidiomycota |
| Valenciniidae |  | Anopla | Nemertea |
| Vannellidae | Vannellida | Discosea | Amoebozoa |
| Veneridae | Veneroida | Bivalvia | Mollusca |
| Viridiraptoridae | Glissomonadida | Sarcomonadea | Cercozoa |
| Warnowiaceae | Gymnodiniales | Dinophyceae | Dinoflagellata |
| Watersiporidae | Cheilostomatida | Gymnolaemata | Bryozoa |
| Xarifiidae | Cyclopoida | Hexanauplia | Arthropoda |
| Xestoleberididae | Podocopida | Ostracoda | Arthropoda |
| Xyalidae | Monhysterida | Chromadorea | Nematoda |
| Xylariaceae | Xylariales | Sordariomycetes | Ascomycota |
| Xystonellidae | Choreotrichida | Oligotrichea | Ciliophora |
| Zosteraceae | Alismatales | Magnoliopsida | Tracheophyta |
